# Single-stranded RNA viruses infecting the invasive Argentine ant, *Linepithema humile*

**DOI:** 10.1038/s41598-017-03508-z

**Published:** 2017-06-12

**Authors:** Monica A. M. Gruber, Meghan Cooling, James W. Baty, Kevin Buckley, Anna Friedlander, Oliver Quinn, Jessica F. E. J. Russell, Alexandra Sébastien, Philip J. Lester

**Affiliations:** 1 Centre for Biodiversity and Restoration Ecology, School of Biological Sciences, Victoria University of Wellington, PO Box 600 Wellington, New Zealand; 2 Pacific Biosecurity, Victoria Link Limited, Victoria University of Wellington, PO Box 1762 Wellington, New Zealand; 3 Malaghan Institute of Medical Research, PO Box 7060 Wellington, New Zealand; 4 School of Engineering and Computer Science, Victoria University of Wellington, PO Box 600 Wellington, New Zealand

## Abstract

Social insects host a diversity of viruses. We examined New Zealand populations of the globally widely distributed invasive Argentine ant (*Linepithema humile*) for RNA viruses. We used metatranscriptomic analysis, which identified six potential novel viruses in the *Dicistroviridae* family. Of these, three contigs were confirmed by Sanger sequencing as *Linepithema humile virus-1* (LHUV-1), a novel strain of *Kashmir bee virus* (KBV) and *Black queen cell virus* (BQCV), while the others were chimeric or misassembled sequences. We extended the known sequence of LHUV-1 to confirm its placement in the *Dicistroviridae* and categorised its relationship to closest relatives, which were all viruses infecting Hymenoptera. We examined further for known viruses by mapping our metatranscriptomic sequences to all viral genomes, and confirmed KBV, BQCV, LHUV-1 and *Deformed wing virus* (DWV) presence using qRT-PCR. Viral replication was confirmed for DWV, KBV and LHUV-1. Viral titers in ants were higher in the presence of honey bee hives. Argentine ants appear to host a range of’ honey bee’ pathogens in addition to a virus currently described only from this invasive ant. The role of these viruses in the population dynamics of the ant remain to be determined, but offer potential targets for biocontrol approaches.

## Introduction

Social insects carry a range of viruses that can have a major effect on host population dynamics. Perhaps the best known viral community is from honey bees, which has been the focus of considerable study due to their economic importance. A range of different factors are likely to contribute to colony collapse and bee declines in general, with viruses frequently considered key players^1, 2^. A recent review noted honey bees host 24 viruses, primarily in the *Dicistroviridae* and *Iflaviridae* families^3^. Of these, the *Deformed wing virus* (DWV) has been suggested as a likely candidate for the majority of global honey bee colony losses during the past 50 years^4^. Such viruses, however, are not restricted to honey bees. There is increasing evidence that these ‘honey bee’ viruses are common in a wide range of insect hosts^5–7^.

Other social insects have been found to carry their own unique suites of viral pathogens. For example, over the last decade four viruses have been described from the red imported fire ant (*Solenopsis invicta*)^8^. These were the first viruses fully described from ants. Three of these viruses are positive-sense, single-stranded RNA (ssRNA) viruses, with one (*Solenopsis invicta virus-1*, SINV-1) assigned taxonomically to the *Dicistroviridae* family, one (*Solenopsis invicta virus-3*, SINV-3) in a proposed new family, *Solinviviridae* ^9^ and the third currently unclassified (*Solenopsis invicta virus-2*, SINV-2)^8, 9^; The fourth virus is a DNA virus, and has been placed in the family *Parvoviridae* ^10^. One of the three ssRNA viruses, SINV-3, shows promise as a biocontrol agent as it can cause significant mortality in laboratory fire ant colonies^11^. Metatranscriptomic and pyrosequencing techniques have proven particularly useful for viral discovery in these and other ant species (e.g. Valles *et al*.^9^, Johanssen *et al*.^12^, Valles *et al*.^13^). Using these and complementary approaches we have found the invasive Argentine ant (*Linepithema humile*) to host a previously undescribed virus *Linepithema humile virus-1* (LHUV-1) and DWV^7^. Replication of both viruses was confirmed within Argentine ants indicating that these viruses were parasitizing their hosts. Our previous work recovered 1,200 nucleotides of the LHUV-1 virus, but did not recover the entire non-structural polyprotein region which would have confirmed accurate phylogenetic placement. In that work we also noted evidence for the likely presence of other novel and known viruses. We found other novel contigs of viral origin (n1905, n1000 and n1050), but were unable to characterise these further.

Argentine ants have been described as one of the six most widespread, abundant and damaging invasive ants^14^. They are a globally distributed pest species with biodiversity, social and economic impacts estimated to annually cost millions of dollars to countries including New Zealand^15^. Population collapse and substantial reduction in range distributions have been previously observed in Argentine ants^16, 17^. Pathogens such as viruses have been hypothesized to be responsible for these collapses^16^. Argentine and other invasive ants are thought to be susceptible to pathogens because of their limited genetic diversity, their high abundance, and a ‘unicolonial’ lifestyle that could facilitate pathogen spread due to ant workers frequently moving between nests^18^. We examined New Zealand populations of this ant to determine the diversity of known and novel RNA viruses that they host using metatranscriptomic approaches. The metatranscriptomic sequencing results were then confirmed, and an indication of the relative infection level for the detected viruses assessed using qRT-PCR. We used Sanger sequencing to confirm and further define the genomes and phylogenetic placement of the novel viruses from these invasive ants. We also comment on the use of multiple metatranscriptomic approaches to recover virus data.

## Results

### Metatranscriptomic virus detection and discovery

**Figure 1:**
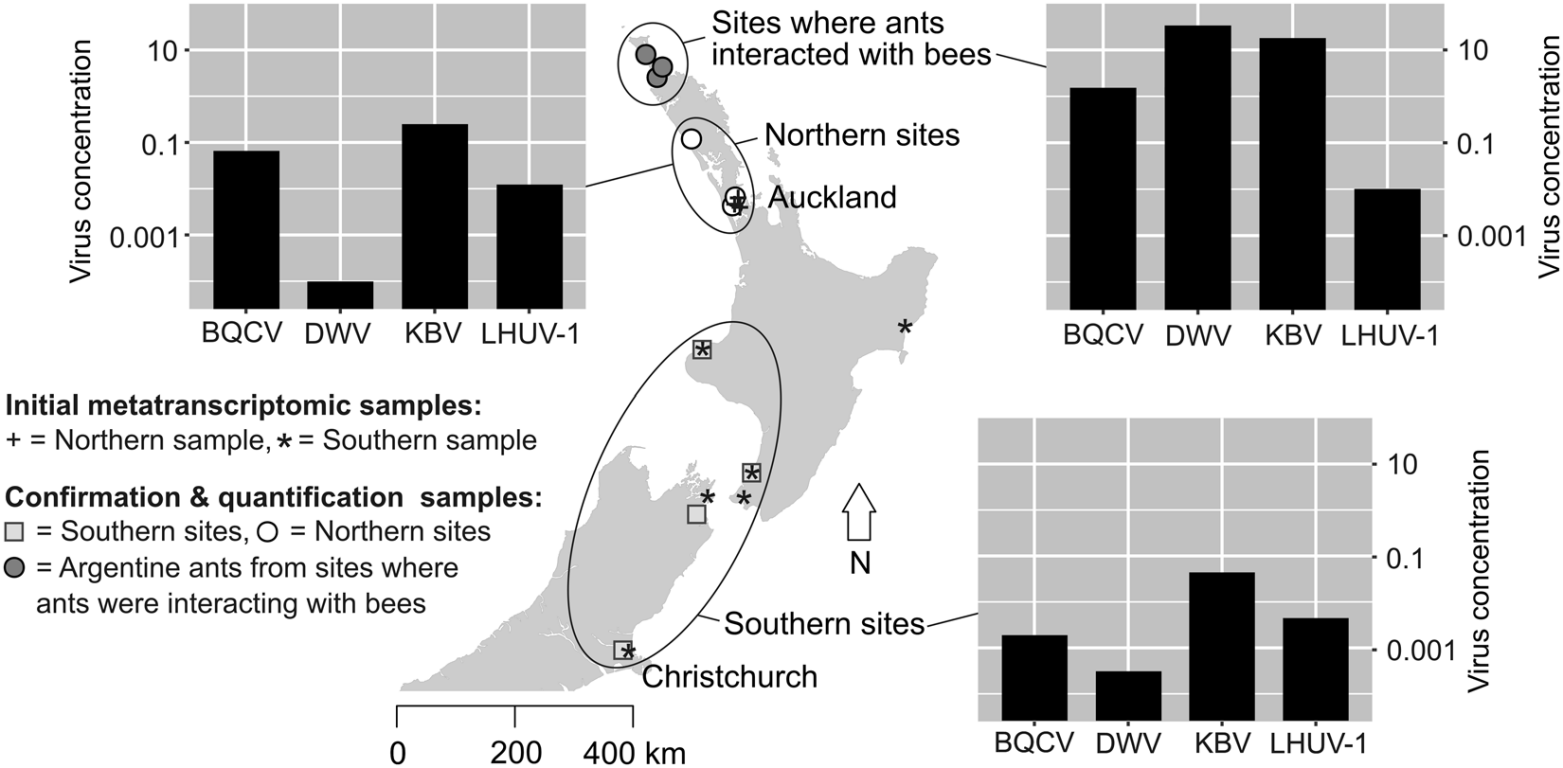
Sampling sites in New Zealand. Both the samples for the initial metatranscriptomic screen (including Sanger sequencing; n = 2) for viruses and the additional samples taken for virus confirmation and quantification (RT-PCR; n = 3) are shown. Samples from the northern-most sites included Argentine ants that were observed raiding honey bee hives, while the other sites were urban and distant from beehives. The concentration of viruses is derived from a standard curve, normalised to the concentration of the internal reference gene *Ndufa8* (and due to the normalisation is unit-less). The map was generated in R v 3.1.1^49^ with the packages ‘maps’^50^, ‘mapdata’^51^,‘maptools’^52^ and ‘GISTools’^53^. Full details of sites sampled are presented in Supplementary Table S3).

**Figure 2:**
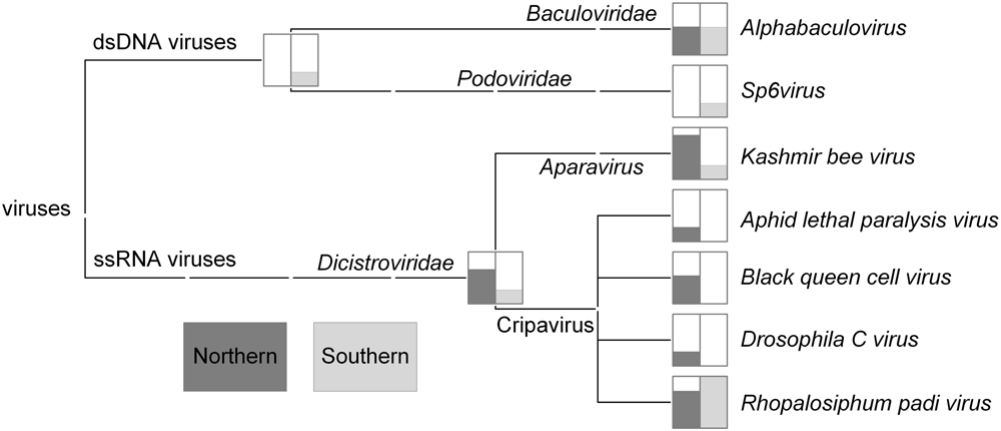
MEGAN taxonomic assignment of metatranscriptomic contigs. Putative viral sequences were based on BLAST similarity obtained using virus blastx matches for samples of Argentine ants from northern and southern samples. The MEGAN results were further refined using Virusfinder, Bowtie 2 and RT-PCR (Table 1).

Our metatranscriptomic detection of viruses and discovery of potential novel viruses used ants that were collected from northern and southern locations in New Zealand (Fig. 1). Illumina RNA-seq followed by assembly with Trinity v 2.0.6^19^ generated metatranscriptomic contigs. The metatranscriptomic contigs were taxonomically assigned to viruses using MEGAN^20^. Our MEGAN analysis determined the new metatranscriptomic contigs to be have a high degree of similarity to the bee viruses *Kashmir bee virus* (KBV), *Black queen cell virus* (BQCV), and *Acute bee paralysis virus* (ABPV), as well as other ssRNA viruses known to affect insects, and two dsDNA viruses (Fig. 2). All RNA virus matches were to members of the *Dicistroviridae* family. Our results revealed differences in composition of matches between the northern and southern samples. In contrast to the northern samples, our southern samples revealed no matches to *Aphid lethal paralysis virus* (ALPV), BQCV or *Drosophila C virus* (Fig. 2). We note that additional sampling would be required to definitively confirm the absence of these viruses from these southern sites and populations.

We identified six putative viral contigs using Blast2GO^21^, based on their similarity to known virus coding proteins (TR30069|c0, TR30069|c1, TR30079|c2, TR31304|c0, TR86646|c0 and TR94176|c0; Supplementary Table S1). To confirm our new contig sequences were not chimeric we designed primer sets to recover their entire length. We could only recover contigs TR30069|c0, TR30069|c1, and TR31304|c0. We recovered 3585 base pairs for TR31304|c0. This sequence matched most closely to BQCV non-structural polyprotein. An open reading frame (ORF) of 1016 codons was predicted by HHpred^22^ to span the viral Helicase protein (100% probability, p < 0.001), and was identical to the Helicase protein for BQCV (GenBank accession ABS82427). For TR30069|c0 1519 base pairs were recovered, which, matched most closely to KBV, IAPV, ABPV and *Formica exsecta 1 virus* (FEX-1) non-structural polyprotein. HHpred predicted an ORF of 313 codons to partially include the RNA dependent RNA polymerase (RdRp) protein (100% probability, p < 0.001) most closely matching KBV. As we could not recover the entire RdRp protein for TR30069|c0 we do not present further results for this contig. TR30069|c1 was recovered in 3 fragments (1360, 590 and 846 bases). GenBank blastp searches matched all fragments most closely to *Israeli acute paralysis virus* (IAPV), KBV, FEX-1 and ABPV. HHpred did not predict functional viral proteins in the longest fragment. The second fragment contained an ORF of 100 codons that HHpred predicted to include part of Protease 3CG (100% probability, p < 0.001). HHpred predicted the third fragment with an ORF of 252 codons to partially span RdRp (100% probability, p < 0.001). The TR30069|c1 RdRp fragment matched LHUV-1 RdRp, and the Protease 3CG matched contig n1905. We concluded the TR30069|c1 contig was chimeric owing to misassembly, and thus excluded it from further analysis.

### Further characterization of LHUV-1 and putative novel viruses

**Figure 3:**
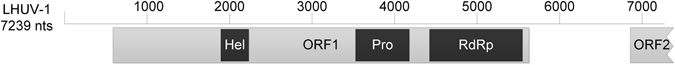
Organization of *Linepithema humile virus-1* LHUV-1 partial genome. The total nucleotide sequence length that was recovered from our analysis is indicated on the left of the figure. The light grey box indicates the predicted open reading frame (ORF1) of 1930 amino acids. Darker grey boxes identify protein motifs and their position within the ORF. The jagged grey box indicates the partially recovered ORF2, which contained a sequence that matched *Dicistroviridae* capsid proteins. Identifiable conserved ssRNA virus protein domains in ORF1 (Hel = helicase [position 549–664], Pro = Protease [position 1153–1381], RdRp = RNA-dependent RNA polymerase [position 1433–1927]) are indicated.

We used the Iterative Viral Assembler (IVA)^23^ to computationally extend the sequence information for LHUV-1 from 1,200 to 8,268 bases, and designed primers for confirmatory Sanger sequencing (Supplementary methods). Sanger sequencing recovered 7,239 bases. None of the newly identified contigs (TR30069|c0, TR30069|c1, and TR31304|c0) or previously identified contigs (n1000, n1050, n1905^7^) aligned with the extended LHUV-1 sequence. The LHUV-1 sequence translated into a single large ORF (Fig. 3), which blastp searches identified as most closely matching the viral non-structural polyproteins (Helicase, Protease, RdRp) of the FEX-1 and IAPV viruses (99% coverage and 50% identity). The entire coding regions of these proteins were recovered. Of the top 100 BLAST hits the closest matches of our LHUV-1 ORF1 and proteins were to the *Dicistroviridae* family. In addition we recovered 164 residues of that most closely matched ORF2 (the structural polyprotein coding region) of *Dicistroviridae* (e.g. KBV, IAPV, ABPV). The ORF2 sequence included the partial sequence of the capsid gene (Fig. 3).

We extended the n1905 contig to 5,840 bases using IVA, which was further extended to 9,450 bases by Sanger sequencing. The resulting sequence matched closely to the two KBV genomes on GenBank (100% coverage and 92–96% identity). The n1905 contig contained a one large (1683 amino acids) and one smaller (843 amino acids) ORF. Like other *Dicistroviridae*, the first ORF of n1905 encodes the non-structural polyproteins (Helicase, Protease, RdRp), and matched the KBV non-structural polyproteins on GenBank with 100% coverage and 99% identity. In *Dicistroviridae* the second ORF codes for the structural polyproteins. Our second ORF coded for capsid polyproteins, and matched KBV ORFs on GenBank with 100% coverage and 98% identity.

Poly(A) tails were not recovered for either LHUV-1 or n1905 extended sequences, indicating that we did not fully recover the genomes of either virus. Based on the published KBV genomes we recovered all but 37 5′ bases and 25 3′ bases (excluding the poly(A) tail). As LHUV-1 is a novel virus we do not know how much of the genome we recovered. However, other *Dicistroviridae* viruses have genomes ranging from 8.5–10.2 kb. Thus we likely recovered at least 70% of the LHUV-1 genome, including the complete ORF1 region (non-structural polyproteins) and part of ORF2. The TR31304, TR30069|c0, TTR30069|c1, n1000 and n1050 contigs failed to extend using IVA, possibly because these were chimeric or fragmented assemblies.

**Figure 4:**
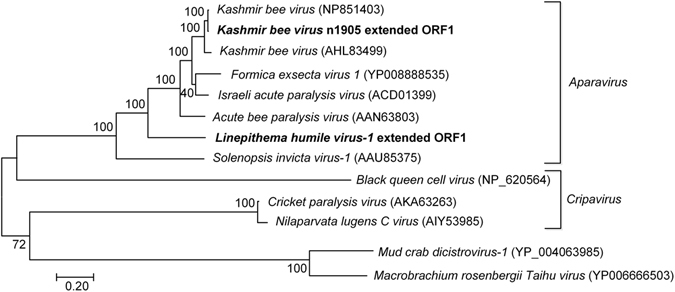
Phylogenetic tree of the Dicistroviridae ORF1 region. The tree includes the extended contig n1905 (proposed KBV strain) and LHUV-1 using a Le & Gascuel (LG)^54^ model with 500 bootstrap replicates. The ORF1 sequences included in the analysis comprise the complete Helicase, Protease and Ribosomal dependent RNA polymerase (RdRp) domains of these viruses and intergenic regions. *Macrobrachium rosenbergii Taihu virus* and *Mud crab discistrovirus-1* (two unclassified *Dicistroviridae* that had ~50% identity to our contigs in blastp results) were included to root the tree. Our sequences are shown in bold text. GenBank accession numbers are shown in brackets.

Our phylogenetic analysis to place the n1905 and LHUV-1 extended contigs within the *Dicistroviridae* family was based on the complete ORF1 coding regions (including the Helicase, Protease and RdRp domains and intergenic regions) (Fig. 4). The n1905 contig ORF1 differed from the published ORF1’s of KBV (GenBank accessions NP851493 [Pennsylvania] and AHL84399 [Korea]) by 27 and 108 amino acids respectively. The published genotypes differ from each other by 107 amino acids. We consider this difference sufficient to propose the n1905 contig as a new genotype or strain of KBV. LHUV-1 was positioned intermediate to SINV-1 (664 amino acid differences) and ABPV (864 amino acid differences) and is thus somewhat distinct from other *Dicistroviridae* (Fig. 4).

**Figure 5:**
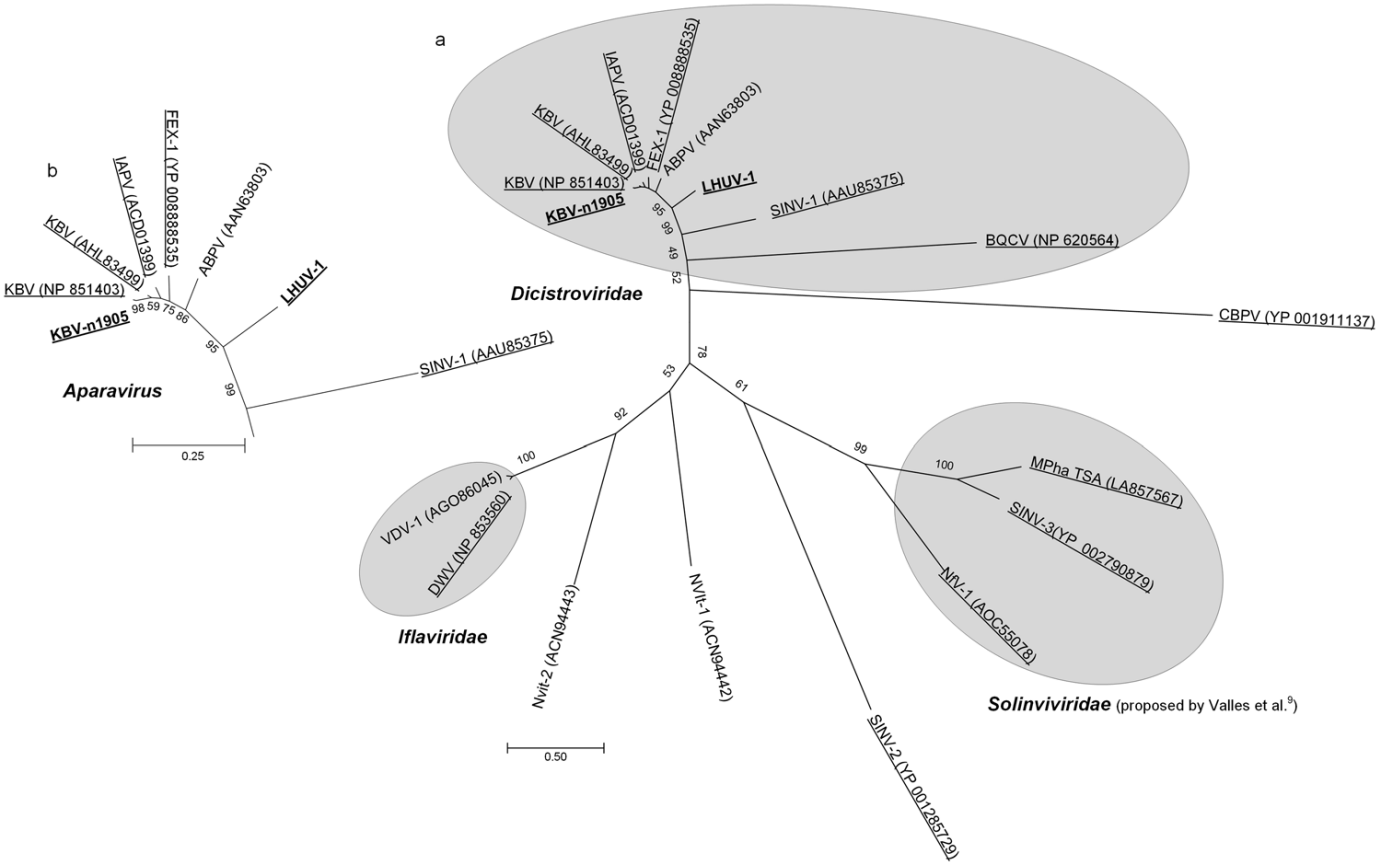
Phylogenetic tree of viruses in ants and other Hymenoptera. The tree (**a**) was inferred using complete Ribosomal dependent RNA polymerase (RdRp) sequences, based on a Le & Gascuel (LG)^54^ model with Gamma parameter and invariant sites (LG+G+I) with 500 bootstrap replicates. A TSA that is a putative virus of the ant *Monomorium pharoanis* ^9^ was also included. Our sequences are shown in bold text. Taxa found in ants are underlined. GenBank accession numbers are shown in brackets. The inset figure (**b**) shows the *Aparavirus* genus of *Dicistroviridae*, with bootstrap support. *Solenopsis invicta virus* 1,2 and 3 (SINV-1, SINV-2, SINV-3); *Formica exsecta virus* 1 (FEX-1); *Deformed wing virus* (DWV); *Kashmir bee virus* (KBV); *Acute bee paralysis virus* (ABPV); *Black queen cell virus* (BQCV); *Chronic bee paralysis virus* (CBPV); *Israeli acute paralysis virus* (IAPV); *Monomorium pharaonis* TSA (Mpha TSA); *Varroa destructor virus 1* (VDV-1); *Nylanderia fulva virus 1* (NfV-1); *Linepithema humile virus 1* (LHUV-1); *Nasonia vitripennis virus 1* (Nvit-1); *Nasonia vitripennis virus 2* (Nvit-2).

Our phylogenetic tree of RdRp sequences for known and predicted viruses of ants and other Hymenoptera assigned LHUV-1 and the new strain of KBV consistently with our tree based on ORF1. The placement of these and other viruses was consistent with the phylogeny of picorna-like viruses^9^ (Fig. 5).

### Additional screening and quantification of known viruses

As well as using MEGAN to screen for novel viruses, we mapped our RNA-seq sequences to selected known ant and bee RNA viruses (SINV-1, SINV-2, SINV-3, FEX-1, IAPV, DWV, KBV, BQCV, ABPV, *Chronic bee paralysis virus* (CBPV), *Varroa destructor virus 1* (VDV-1) and *Sacbrood virus*) using Bowtie 2^24^ and to all known viruses using Virusfinder^25^. To determine whether we had contamination we also mapped our negative control sequences, of which a single sequence Bowtie 2 mapped to FEX-1. Our sample sequences mapped most closely to KBV, with fewer matches to FEX-1, IAPV, ABPV, BQCV and SINV-1 (Table 1). Virusfinder produced similar results but detected only ABPV, CBPV, IAPV and KBV sequences.

**Table 1:** Detection of known viruses from RNA-seq reads using MEGAN, Bowtie 2 and Virusfinder, and confirmation using RT-PCR. The MEGAN figures indicate the number of contigs detected for each given virus.

Virus	MEGAN	Bowtie 2		Virusfinder		RT-PCR	Replication
	Argentine ant samples	Argentine ant samples	Negative control	Argentine ant samples	Negative control		
*Alphabaculovirus*	8	—	—	N	N	—	—
*Sp6virus*	2	—	—	N	N	—	—
*Aphid lethal paralysis virus*	2	2	N	N	N	—	—
*Drosophila C virus*	2	N	N	N	N	—	—
*Rhopalosiphum padi virus*	21	N	N	3 (47)	N	—	—
ABPV	N	424	N	2 (368)	N	N	—
BQCV	4	127	N	4 (2,273)	N	Y	N
CBPV	N	N	N	N	N	N	—
DWV	N	1	N	N	N	Y	Y
FEX-1	N	2,419	1	N	N	N	—
IAPV	N	1,012	N	5 (2,172)	N	N	—
KBV	11	18,682	N	2 (40,343)	N	Y	Y
*Sacbrood virus*	N	N	N	N	N	—	—
SINV-1	N	14	N	N	N	N	—
SINV-2	N	N	N	N	N	—	—
SINV-3	N	N	N	N	N	—	—
VDV-1	N	N	N	N	N	N	N
LHUV-1	N/A	N/A	N/A	N/A	N/A	Y	Y

The figures reported for Bowtie 2 are those reads that aligned concordantly 1 or more times or discordantly 1 or more times. Virusfinder results show the number of contigs (reads) that mapped to the virus. The RT-PCR column indicates if the virus was detected using RT-PCR, and the Replication column indicates whether replication of the virus was confirmed subsequent to RT-PCR. In each of the columns ‘-’ indicates the virus was not tested for and ‘N’ indicates non-detection. *Acute bee paralysis virus* (ABPV); *Black queen cell virus* (BQCV); *Chronic bee paralysis virus* (CBPV); *Deformed wing virus* (DWV); *Formica exsecta virus* 1 (FEX-1); *Israeli acute paralysis virus* (IAPV); *Kashmir bee virus* (KBV); *Solenopsis invicta virus* 1, 2 and 3 (SINV-1, SINV-2, SINV-3); *Varroa destructor virus 1* (VDV-1), *Linepithema humile virus 1* (LHUV-1). LHUV-1 was not detected by MEGAN or Virusfinder as the sequence was not lodged in GenBank at the time the study was undertaken.

We used qRT-PCR to screen samples for the presence of viruses we had detected using Bowtie 2 and Virusfinder, together with honey bee viruses that have been recorded previously in ants. The presence of KBV, BQCV, LHUV-1 and DWV was confirmed by qRT-PCR (Fig. 1). Ants from sites where ants interacted with bees had the highest viral loads. Where ants did not interact with bees, we found a lower prevalence of DWV relative to other viruses. LHUV-1 viral loads were similar among all sites. We found no difference in viral loads between northern and southern sites. Viral replication was confirmed for three of these four viruses (LHUV-1^7^, DWV, and KBV). No amplification was observed in RT-PCR for ABPV, CBPV, IAPV, VDV-1, SINV-1 or FEX-1.

## Discussion

The first virus was described from ants only 13 years ago^8, 26^. Since that publication next generation sequencing approaches have been used to discover other viruses, but only for a limited number of ant species and locations^7, 9, 10, 12^. Our study has demonstrated that the invasive Argentine ant hosts a range of viruses. Our metatranscriptomic analysis detected six novel putative viral contigs, three of which we were able to partially or fully recover by Sanger sequencing. Of the putative novel viruses for which we recovered extended sequences, one (which partially matched the previously detected n1905 contig^7^), was resolved as a new strain of KBV. The second matched the LHUV-1 sequence when recovered by Sanger sequencing, and the third matched BQCV. We extended the known sequence of the Argentine ant LHUV-1 virus to approximately 70% of the genome. Our qRT-PCR results detected BQCV, KBV, LHUV-1 and DWV, of which viral replication (i.e. parasitism by viruses of the ants) was confirmed for all but BQCV. A lack of observed replication for BQCV may indicate that these ants are not true hosts for this virus and it is not parasitizing them, or that the method used was not sensitive enough to detect replication at low virus titers (abundance). However, BQCV might replicate in Argentine ants under conditions or at time points missed in our survey. Viral replication does not occur at all times in all infected hosts^27, 28^.

Our results demonstrate that populations of the invasive Argentine ant in New Zealand host a range of RNA viruses. As well as the Argentine ant LHUV-1 virus, the ants also host viruses that are associated with disease in honey bees, including KBV and DWV. These are globally widespread and important viruses. DWV, for example, has been hypothesised as the most likely candidate for the majority of global honey bee colony during the past 50 years^4^. These viruses are clearly also parasitizing Argentine ants, as replication was confirmed. While DWV has been detected in Argentine ants previously^7, 29^, our study is the first record of these or any ants hosting KBV (and possibly BQCV).

Although the abundance of viruses (titer or viral load) such as LHUV-1 and DWV in Argentine ant populations in New Zealand is known to vary over time, the viruses appear to be consistently present in these ant populations^29^. In areas where these ants are associated with honey bee colonies, the honey bees appear to be infected with substantially higher DWV loads. In honey bee populations, viruses like DWV and KBV are associated with colony collapse, but only when viral load is high^30^. In New Zealand, another study demonstrated that the presence of Argentine ants was associated with higher DWV loads in honey bees and an average 47% mortality of hives over a 6-month period^29^. The same DWV strain was observed in both Argentine ants and bees. Argentine ants appear to have the dual effects of substantially elevating DWV titers in bees and raiding their hives for honey and brood^29^. We found that viral load of both DWV and KBV was also higher in Argentine ants when these ants were interacting with bees: markedly so for DWV, with viral concentrations five orders of magnitude higher when the ants were present in honey bee apiaries. These viruses were also present at all other sites, including in urban areas where there were no bee hives with several hundred meters of the sample collection location. Viruses such as DWV, KBV and BQCV are clearly present in these invasive ants in the absence of close contact with bees.

Some researches might suggest that the viruses we detected could alternatively or additionally be infecting bacterial symbionts or pathogens within Argentine ants. Bacteriophages, however, tend to belong to specific families and none have been previously found in the *Dicistroviridae* family. Viral infection of bacteria within Argentine ants thus seems highly unlikely. Although these viruses are clearly parasitizing Argentine ants (or possibly their endosymbionts), as shown by active replication, we do not know what their effect is on ant colonies, and studies have yet to investigate the effects of honey bee viruses on ant population dynamics. We hypothesise, however, that these viruses contribute to the population collapse of Argentine ants in New Zealand and elsewhere^18^. Possibly these viruses remain in a latent state until environmental or other conditions stimulate virulence. If so, it could be possible to induce virulence as a novel biocontrol, potentially by compromising the immunity of these ants.

Other studies have suggested that interactions between other host species result in honey bee viruses being shared between many Hymenopteran and non-Hymenopteran hosts (e.g. Levitt *et al*.^6^, Furst *et al*.^31^). DWV has previously been found in *Camponotus* ant species, together with IAPV^6^ and CBPV^32^, which was also hosted by the ant *Formica rufa* ^32^. No ant viruses have been reported from honey bees, although this may be due to few studies specifically examining honey bees for ant viruses. To our knowledge honey bees have only been examined for LHUV-1 and SINV-3^29, 33^. Moreover, host specificity tests of SINV-3 have found that it does not infect ant species other than *Solenopsis invicta*, including closely related ant species^34^. Viruses continue to remain under-studied outside of model organisms^35^, and their prevalence within species, and degree of inter-specific transmission is still not well understood.

We detected a novel strain of KBV by extending a contig that previously appeared likely to be a novel ant virus^7^. We recovered a total of 9,450 bases of this KBV strain, allowing definitive phylogenetic placement. Our data matched closely to the two KBV genomes on GenBank, but differences in ORFs indicate this is a new strain of the virus. KBV has not previously been reported in ants, although it is a common and globally widespread virus of honey bees^36^. One study indicated between 30–40% of New Zealand honey bees are infected with KBV^37^. Another New Zealand study found a 100% KBV infection rate in six different nests of common wasps (*Vespula vulgaris*)^38^. KBV was not detected in our earlier study on Argentine ants using RT-PCR^7^, although it appears from our current results to be highly prevalent, particularly where ants interact with bees. The lack of detection in earlier work may be due to the differences in the RT-PCR primers used between studies not being able to detect the proposed new strain. If this is the case, variant strains of KBV might exist undetected in ant (and potentially honey bee) populations. We know that some virus strains are more virulent than others^39^. But we do not know which KBV strains are present in New Zealand honey bees or the distribution of the new strain globally, their relative virulence, and the degree of transmission between species.

We extended the known genomic sequence of the LHUV-1 virus to cover approximately 70% of the estimated genome. Our phylogenetic analysis of both the entire ORF1 and RdRp gene clearly place LHUV-1 as an *Aparavirus* in the *Dicistroviridae* family, phylogenetically positioned intermediate to ABPV and SINV-1. ABPV and SINV-1 have been found to be similarly related in other work using amino acid sequences of the capsid proteins (e.g. Tufts *et al*.^40^). We recovered ORF1 for LHUV-1, which included all viral non-structural coding regions. We have thus characterized the virus sufficiently to confirm its identity as a novel virus infecting Argentine ants. Our previous work has indicated LHUV-1 to be present in the ant’s home range of Argentina, and in the invaded range of Australia and New Zealand^7^. The virus is thus likely to be distributed throughout the ant’s invaded range.

The ssRNA viruses that to date are known to be hosted by ants typically occur within three viral families: *Iflaviridae* (DWV^6^); *Dicistroviridae* (SINV-1^26^, LHUV-1^7^, KBV - this study, IAPV^6^, and possibly BQCV - this study); and *Solinviviridae* (SINV-3, *Nylanderia fulva virus 1*, and a potential virus infecting the ant *Monomorium pharaonis* ^9^). The SINV-2 virus has not been placed within a family, but like the other viruses associated with ants, it is a picorna-like virus^9^. Similarly, CBPV, which has also been found in ants^32^ is currently an unclassified ssRNA virus. Perhaps this apparent phylogenetic restriction to a few families is owing to a co-evolutionary history. However it might also be due to a lack of studies, or the ease of detection of ssRNA viruses relative to other viral groups.

We used complementary methods in our virus discovery and confirmation in order to maximise our ability to detect potential novel viruses. We found that IVA^23^ effectively enabled us to extend the sequence length of putative novel viruses and strains. We could then use this sequence as a template for confirmation of presence via standard PCR and Sanger sequencing. Typically, RACE is used to extend these sequences (e.g. Oi *et al*.^9^). RACE can be expensive compared with standard PCR^41^. Thus our approach offers a cost effective alternative, particularly for narrowing the list of candidates for confirmation. While our approach enabled recovery of nearly an entire novel strain, in order to recover a full novel genome, some RACE or similar sequencing approach (e.g. Dallmeier & Neyts^41^) will most likely be needed.

We used Bowtie 2^24^, Virusfinder^25^ and MEGAN^20^ to identify known and novel viruses. Although Bowtie 2 and Virusfinder indicated the potential presence of more bee viruses than MEGAN, the latter results were more consistent with qRT-PCR confirmatory analysis. These differences in results can likely be attributed to the difference in analysis approaches: MEGAN analysis is based on blast results of searches on GenBank databases using assembled contigs, while Bowtie 2 and Virusfinder use individual RNA-seq reads to map entire known genomes. We therefore advise caution when using single in-silico analyses to make definitive conclusions.

One inconsistency continues to baffle us. Neither this study, nor our earlier work^7^ detected DWV using RNA-seq data. This virus was not observed in any assembly, or in our MEGAN analysis, or in mapping (with the exception of one single read). However, DWV has been positively confirmed in these samples via qRT-PCR here and in other species^7, 29^. We know that this is not a general failure of RNA-seq to detect DWV as we detected this virus in RNA-seq data of honey bee samples that were sequenced in the same run as our ant samples, as others have also found (e.g. Cornman *et al*.^42^). For the same reason this false negative result cannot be attributed to a biological feature of the virus that would make it undetectable by RNA-seq. Our qRT-PCR results showed a much lower prevalence of DWV in sites where Argentine ants were not present with bees, so it seems most likely that the virus was at undetectable prevalence in our current and earlier RNA-seq samples (which were from sites where ants and bees did not interact).

Ant populations are known to undergo substantial fluctuation and even local population extinction^18^. The current study and other publications demonstrate a substantial microbiota associated with Argentine ant populations^7, 43^. Several of these pathogens can substantially influence the population dynamics of other social insects, such as the involvement of DWV and KBV in collapses of honey bee populations^42, 44^. However, it remains to be determined if the viruses we observed have any involvement in the local collapse of Argentine ant populations in New Zealand^16^ or elsewhere^17^.

## Methods

### Metatranscriptomic virus detection and discovery

We used two pooled groups of ants for the metatranscriptomic detection of viruses and discovery of potential novel viruses. In November 2014 we sampled a northern population (Auckland: three sites), and in November 2014 and June 2015 we sampled a southern population (six sites: Paraparaumu, Picton, New Plymouth, Christchurch, Petone, Gisborne) (Fig. 1; Supplementary Table S3). Ants were collected in RNA*later* Stabilization Solution (AMBION Inc., Austin, USA). RNA was extracted from pools of whole ants from the northern (150 ants) and southern populations (120 ants). Purification of total RNA was carried out using a PureLink RNA Mini Kit® (Thermo Fisher Scientific Inc., Waltham, USA) according to the manufacturer instructions, after homogenisation with a Qiagen Tissuerupter (QIAGEN, Tokyo, Japan). RNA integrity was confirmed and quantified with an RNA 6000 Nano chip on the Agilent 2100 Bioanalyser (Agilent Technologies Co. Ltd., Diegem, Belgium), according to the manufacturer’s instructions. The two pooled samples together with a negative control were sequenced as 125 base paired end barcoded Trueseq libraries on an Illumina Hiseq by New Zealand Genomics Limited (NZGL) at the University of Otago. Post-processing at NZGL included quality control, excluding bases with quality scores Q > 30 and trimming of adapters. We assembled the RNA-seq data using Trinity v 2.0.6^19^ using read normalization.

To detect potential novel viral pathogen sequences in the assemblies, we used the Trinity assembled contigs to query the National Center for Biotechnology Information (NCBI) GenBank databases using BLAST^45^. The nr and nt databases were downloaded from NCBI GenBank (ftp://ftp.ncbi.nlm.nih.gov/blast/db/) on 1 December 2015. BLAST searches were run using an installation of BLAST v 2.2.25, on the Victoria University of Wellington Science Faculty’s High Performance Computing Facility. We used blastx searches to identify putative viral proteins. Searches of the nr database are time consuming for a large number of input sequences, and generate large amounts of data when the default xml output format is used. As we were specifically targeting viruses, and the majority of sequences were likely to be non-viral, we pre-processed the data using blastn searches of the NCBI Genbank nt databases to include sequences that matched virus and viroid taxa, which we output in tabular format. We restricted searches to a threshold e-value of 0.0001 and 90% percent identity or better to enhance search specificity. Taxonomic information was assigned to the tabular output using a custom Perl script (https://github.com/AnnaFriedlander/taxon4blast). From the resulting assignments we filtered the input data for virus taxa using a Perl script. We then used these filtered contigs as input for a blastx search of the nr database with searches restricted to a threshold e-value of 0.001, percent identity of 90, best_hit_overhang of 0.25 and best_hit_score_edge of 0.05. The blastx results were visualised in MEGAN 5.10.7^20^. To confirm these as virus candidates we identified coding sequences of novel viral origin in our contigs by visualising our blastx results in Blast2GO^21^, and translating these contigs using ExPASy (Bioinformatics Resource Portal, Swiss Institute of Bioinformatics, http://web.expasy.org/) to determine the appropriate reading frame.

Six contigs were identified as potential novel viruses (Supplementary Table S1), based on their similarity to known virus coding proteins. We excluded TR86646|c0 and TR94176|c0 contigs at this point as they were relatively short sequences (529 and 619 bases respectively) and less likely to yield further informative data. To confirm the remaining contigs (TR30069|c0, TR30069|c1, TR30079|c2 and TR31304|c0) were not chimeric due to misassembly we designed overlapping primer sets to recover the entire length of the contigs. These were Sanger sequenced, then checked and aligned using MEGA7^46^. Reliable sequences could only be retrieved for TR30069|c0, TR30069|c1, and TR31304|c0. We translated the sequences using ExPASy and used GenBank blastp searches within MEGA7 to identify the most closely matching viral proteins.

### Characterization of LHUV-1 and putative novel viruses

We used IVA^23^ to extend the sequence information for the LHUV-1 virus using the RNA-seq data, and aligned the newly identified contigs (TR30069|c0, TR30069|c1, and TR31304|c0) and previously identified contigs (n1000, n1050, n1905^7^) with the extended LHUV-1 sequence in MEGA7. We also used IVA to extend the newly identified and previously identified contigs, and Sanger sequenced those that could be extended (Supplementary Table S2). For n1905, which appeared to closely match KBV, we extended the 3′ end using primers based on the KBV genomes published in GenBank.

We translated the extended contigs using ExPASy and identified the most closely matching viral proteins with HHpred^22^. We created a maximum likelihood phylogenetic tree for the n1905 and LHUV-1 ORF1s, together with other *Aparavirus* complete ORF1 sequences from GenBank to determine the placement of our sequences within the group. Finally, we created a single phylogenetic tree from complete Ribosomal dependent RNA polymerase (RdRp) sequences together with sequences from GenBank and one TSA sequence predicted to be a virus of ants and other Hymenoptera (Supplementary Table S1 in Valles *et al*.). We restricted the analysis to ants and othe Hymenoptera as these groups were the closest matches to our protein sequences on GenBank. We translated the TSA sequence to obtain the RdRp region for comparison. Sequences were aligned using MUSCLE^47^ and phylogenetic trees were constructed in MEGA7.The alignments for both phylogenetic analyses are provided in the Microsoft Excel spreadsheet associated with Supplementary Table S5.

### Additional screening and quantification of known viruses

As well as using MEGAN to screen for all viruses, we screened our sequences for viruses known to infect bees and ants by mapping the RNA-seq reads to those virus genomes using Bowtie 2^24^ and Virusfinder^25^, which uses the nr database as a reference, to identify all known viruses. For all putative viruses detected by MEGAN, we mapped the RNA-seq reads directly to their viral genomes using Bowtie 2 to identify potential true and false positives. We then used Virusfinder to also identify potential false positives and false negatives. We then used RT-PCR to confirm the positive results from Bowtie 2 and Virusfinder. We also used RT-PCR to confirm false negatives and true negatives using selected viruses, and confirm true positives and true negatives of the viruses identified by Virusfinder and Bowtie 2 (Table 1). Viruses screened with Bowtie 2 included the ant RNA viruses: *Solenopsis invicta virus* 1,2 and 3 (SINV-1, SINV-2, SINV-3), *Formica exsecta virus* 1 (FEX-1),and viruses commonly infecting honey bees: DWV, *Kashmir bee virus* (KBV), *Acute bee paralysis virus* (ABPV), *Black queen cell virus* (BQCV) and *Israeli acute paralysis virus* (IAPV), *Chronic bee paralysis virus* (CBPV), *Varroa destructor virus 1* (VDV-1) and *Sacbrood virus*.

Additional samples of Argentine ants were collected to both confirm the presence and compare the relative infection level of identified viruses (Fig. 1). Viruses were detected and loads were quantified (where applicable) in Argentine ant samples using qRT-PCR for KBV, LHUV-1, BQCV, DWV, and RT-PCR for ABPV, CBPV, IAPV, SINV-1 and FEX-1. Three composite samples were used in this analysis: a sample from southern sites (Blenheim, Christchurch, New Plymouth, and Paraparaumu), a northern sample (two sites in Auckland, and one in Dargaville), and three sites in Northland where Argentine ants were found to be attacking bee hives (Rangiputa, Te Kao, and Waipapa) (Fig. 1; Supplementary Table S3). All ants were collected alive and snap frozen at either −80C or in liquid nitrogen.

RNA was extracted from 24–30 whole ants from each site by bead-beating (BeadBeater 16, Biospec Products, USA) samples in GENEzol reagent (Geneaid, Taiwan) with 5% β-mercaptoethanol, with chloroform and isopropanol purification. These extractions were then pooled to provide RNA for the northern, southern locations, and where Argentine ants were co-located with bees (Supplementary Table S3). Concentrations of RNA were quantified with a NanoDrop spectrophotometer (Thermo Fisher Scientific Inc., Waltham, USA). A 1 µg/sample was used for cDNA synthesis using qScript XLT SuperMix (Quantabio, Beverly, USA). Samples were then analyzed in duplicate by RT-PCR and qRT-PCR with PerfeCTa SYBR Green reagent (Quantabio, Beverly, USA) using 1 µl cDNA/reaction using published and designed primers (Supplementary Table S4). A QuantStudio 7 (Applied Biosystems/Thermo Fisher Scientific, USA) was used for the qRT-PCR with fast cycling conditions and fluorescence detection during the elongation step (Stage 1: 95 °C, 30 s; Stage 2: 40 cycles of 95 °C, 5 s; 60 °C, 15 s; 72 °C, 20 s). Quantification cycle (Cq) values were used to calculate viral loads via external standard curves generated for the viruses and internal reference genes. The external standards were 150–156 base DNA fragments (Thermo Fisher Scientific Inc., Waltham, USA and Sigma-Aldrich, St. Louis, USA) that matched the region recognized by the primers. The internal species specific reference gene *L. humile Ndufa8* (NADH dehydrogenase [ubiquinone] 1 alpha subcomplex subunit 8) was used to normalize the calculated viral loads^48^.

Viral replication was confirmed by detection of the virus negative strand using reverse transcription and RT-PCR based on the standard method for detecting DWV replication^28^. Reverse transcription (SuperScript IV, Invitrogen/Thermo Fisher Scientific, Waltham, USA) of 1 μg RNA was performed with a tagged forward primer at a final concentration of 100 nM (DWV Tag-F15; KBV Tag-KBV83F; VDV-1 Tag-VDVqF [as an additional negative control], or BQCV Tag-BQCV-sense; Supplementary Table S4). PCR was then carried out on the cDNA or no-template negative controls using the Tag primer (agcctgcgcaccgtgg) and the corresponding reverse primer (DWV B23; KBV KBV161R; VDV VDVqPCR-rev, or BQCV BQCV-antisense; Supplementary Table S4). The products were then resolved by 2% agarose gel electrophoresis. Positive controls for DWV and KBV were included (BQCV and VDV positive controls were not available). LHUV-1 replication was confirmed in our earlier study^7^.

## Electronic supplementary material

Supplementary materials and methods

Alignments
