# Supplementary material for: Single-stranded RNA viruses infecting the invasive Argentine ant, Linepithema humile
Source: Sci Rep. 2017 Jun 12;7:3304. doi: 10.1038/s41598-017-03508-z (PMC5468335; doi:10.1038/s41598-017-03508-z)
Supplement: Supplementary file 1 — Supplementary materials and methods [file 41598_2017_3508_MOESM1_ESM.pdf]

Single-stranded RNA viruses infecting the invasive Argentine ant, *Linepithema humile*

Monica A.M. Gruber[1,2]*†, Meghan Cooling[1,2], James W. Baty[1,3], Kevin Buckley[4], Anna Friedlander[4], Oliver Quinn[1], Jessica F.E.J. Russell[1], Alexandra Sébastien[1], Philip J. Lester[1,2]†

1 Centre for Biodiversity and Restoration Ecology, School of Biological Sciences, Victoria University of Wellington, PO Box 600, Wellington, New Zealand

2 Pacific Biosecurity, Victoria Link Limited, Victoria University of Wellington, PO Box 1762, Wellington, New Zealand

3 Malaghan Institute of Medical Research, PO Box 7060, Wellington, New Zealand

4 School of Engineering and Computer Science, Victoria University of Wellington, PO Box 600, Wellington, New Zealand

∗ Monica.Gruber@vuw.ac.nz

† These authors contributed equally to this work

Supplementary Table S1: Putative novel viruses initially detected via metatranscriptomic analysis using MEGAN. The choice of reading frame (RF) and direction was based on the translation that included the highest number of contiguous coding proteins. The blastp result indicates accession number, organism, percentage cover and percentage identity. Note that these were preliminary detections and the final protein coding sequences were derived once Sanger sequencing had recovered further genomic data.

contig	nucleotide sequence	RF & →	putative protein sequence	blastp result
TR30069| c0	GCTCCTAGCCGATGAAGTATCCTGAGCCATGGGGGTATCGATCCTATTTGGAGTTTCCACATCATGAAAGGTGGTGATTTGTTCCTTTTCAATG<br>GCATTCTCCGCTGAGGTCGACGCGAGTTCCGTGTTGTGTACGTTGGTAGAATTGTTGTTTTCTTGGTTATCAGCAGGTATGGTATTTCGAAACG<br>CCGAATATGTTACTATCCGACTGCTATAGCTACCCGATATAGCGGTTTCTATCCAATAGAGGATTCACTGGGGCTGCCAGCTAGGCTCCTCACC<br>CTAAATAGGGCACCTAGACCACTGTTTCAAGCCTTACATTCAAGTTTATTGGAAATTTCCTCAAATGATGGGTGACCATGAAACAGTGCATCTTA<br>AATACTACATTTACATGTAATACTCAAGATACCGCTCCTGAGCATATCCACGATAAGTGTTTATGTCCAGGTGATCTCCTGTAGCCTTTGCATAA<br>GCTTTGTCAATTACTTTTGACCACTTATCAAAGACTCTCTCTTCATGCATGGAAAGTTCCATTATTGCATTTTCACAATTCAATTTAGTACCTTCTT<br>GGATATCGAGTCCTCCTCGACACCAATTGGGCATTTCAAGAATTGTATCCATACATAATGGAGCTTCCCACACCTTCCGTTTATTGTCATATCTA<br>AACTTGCGTTTAAGATATTGCACGTCCTGTATTGATCGCCAGTTTGGTACCTCGCCTCCAACTCCCTTGAGTTCATCTGTATAAGTGAATCCAAG<br>TGTTGAAAAGGCCTCTGCGATAGTAGACATGTTATACCATGCACTAACTTCATCACTGAAGTTAATTACATTATCATCTCCATAAGAAACGAGGG<br>AAACATGTTTACTAAAATCTCTCATCGTCATTTTAACTGCCGCCTTAGTAGCACAGATTGAAAAACACATTCTCAATCCCATACTATTAATAAAGC<br>AGTTAAGGGGTGTCGTAGCGGGGTTTCCTGAGGGTTGGCTATGAGTCATCATGTATACTGAATCATTGCAGATGTGCACAGAATTATACACATC<br>CATCAGCAAAACATGACGTATCAAAGCATTTTCCGGTCCGTCATCGTAAAACTCATTAGCTAAATCTGCAAATTTTTCCATAATGCAAACGTTTAG<br>TGATCCATCAAAAGTTGAAAAATCACCAGCAATTACTTTATTTCCATATTTGGTTAATTTCCTAACAGTTTTACTCCAGTCTTGTGAGTAAACGTTC<br>GTTCCAATAGACACTTCATTTGTAATTCGATTTTCCATCAGATGAGCTATAAAGCCCAAATAATACATTCGAAAAGCAATTGAGAAGTCCATTGGT<br>CCATTTGAGAACACTCGTG	Frame 3 3’-5’	RVFSNGPMDFSIAFRMYYLGFIAHLMENRITNEV<br>SIGTNVYSQDWSKTVRKLTKYGNKVIAGDFSTF<br>DGSLNVCIMEKFADLANEFYDDGPENALIRHVLL<br>MDVYNSVHICNDSVYMMTHSQPSGNPATTPLN<br>CFINSMGLRMCFSICATKAAVKMTMRDFSKHVS<br>LVSYGDDNVINFSDEVSAWYNMSTIAEAFSTLGF<br>TYTDELKGVGGEVPNWRSIQDVQYLKRKFRYD<br>NKRKVWEAPLCMDTILEMPNWCRGGLDIQEGT<br>KLNCENAIMELSMHEERVFDKWSKVIDKAYAKA<br>TGDHLDINTYRGYAQERYLEYYM-M-<br>YLRCTVSWSPII-GNFQ-T-M-GLKQWSRCPI-<br>GEEPSWQPQ-<br>ILYWIETAISGSYSSRIVTYSAFRNTIPADNQENN<br>NSTNVHNTELASTSAENAIEKEQITTFHDVETPN<br>RIDTPMAQDTSSARS	AHL83499.1, KBV non-structural polyprotein, 72, 95
TR30069| c1	ATGAAACTCAATAACTAGTACTAATTCTTAAATAATTTAATCATGATATAAATGCTGAAGACAAGTATTTCGATTTATATCCTCTGAATTTATTAAAG<br>AATATGTAATTACTCGGTGGTGATTACATTTTAGTCGCAGTTAACCTGCAGTTGTAGAGCATCCTGTGTCGGAGCAGTAGTAATGCAACCTATCA<br>ACGAAACATGAACACTTTTAATAAACAACCCGCAACTCTTAAATACCAAGCAAGCAAGAAAATCTTTACCGGAAAACATGATGAACTTCATGTTAT<br>TTGGGAAGATATGATTAGAAGCGCAGTAGAAGACAAACATGTCTATAATTCAACAACATCTTACGCCTTTAATCGAGCATGGTGTTGCTGTACCC<br>GCAAGGAAACAGCAACATTATTTGGAGACTTTTCATGCAATTATTTCCAGTTAACACCATACTGGGAAGGTGAAATGAATGATTATACGGACCTA<br>AATTGTGACCCAATTGAGAACACTATCATGGCATTGCAAATGAGTGAATTTGAACCTATTTGGTTTTACTTACTTAATAAAGTTTTAGAAAGATCA<br>ATACTTGAACAAGACTACCAACATCAGAACTATTGTTTTAAATACATATGTAAAATAAAAGTTTCTGTACATTGGTGGGAAGTTTTAGCACGATCT<br>GGATTCTTTGTTAAGTTAAGTAAATGCCTTTCAGTAGGAGCAATGAAACGACTTTTGCAAAAATGTGGTGATATTGAACTCAATCCCGGACCCAC<br>TTATAAGGAAATTTGCCAGCGCAAATTTAAACGTAACCTTCATTCAGACAAACGAGAAGAATGGAAAGCCCAAAAGCAGATTGAACGACAAATAA<br>GATTAGAAGAAGAAGAGTATGTTCCAAAGAACCATAAAAAGATAATTAAAGAAGTTGAGATGCAAATGTTTGGAACTTTAGGCACTCTTGCCAGT<br>ATTACTGGCAACATGTGGCTGGGAGCAAAAACAAACAACGCATTAGATAAAGCTACGAAATCAATTGATACAGTTACAACCGAAATAACGAAAA<br>CATTGGATCAGTTTAGATTGACTTTTGCTAATTTTAGTGGTATGATGTTTAAATCATTTAATATATTGGATATTATTTCTGATATAATATTTGCTTTA<br>TTACAACTATCATACGCTAAATATGAATGCAAAATTAAATCTTTAACTGTTGAATTTGTTAGAATTATGAGGAAGTACGGATTTGGAATCAGTATC<br>GATTATATTCGCAACCTAATAATGCGACATAGACAACCAAGCATGCAAATTGACGGAAAAATTGACTTTTATGAGTACTTAAACCCAACAACCAT<br>TGTTTCTTTACTTTTTGTTGTGTTATCTACGGTATTTACAACCGTTTTACCAAAGAAAACACACTTAGAAGAAGTAATTAAACGATGTGGGGAGTT<br>AGGACGCTCTTCCAAAGGAATTAAAGACCTGAATGATACTTTACACGAGAGTGTAGGTTTCATGTTAGAATATTTTAAAGTTAACGTTTTAGGATT<br>AACCCCAACAGAAAAGATAGAGGAATTTATTAGTGGTATCGACCGCTGGTTTGATGATGTAAGAAATTTCCTGCAAAGGAAAGATTTTGCTGATA<br>AAAAATCAGAAGATATCATGAAAGATCCCAATGTCTTAGTAGAAGTAGAAAATATATATAAGCGCGGATTGGAGTTTTCGCGCGAAATTTCCGAT<br>AAGAAACTCCAAAGAGAATTGACCCTACCATTCCAGACTCACATGAAGTATGTGACAGATTTAATGAAACTAGTTGACACTAGTGGAGCTTTTGG<br>TTCGCGTCCTAGGACGCAACCAATTGTAATCTGGCTATACGGAGAGTCAGGAGTTGGCAAATCAGGAATGAGTTGGCCACTTGCAGTGGATTT<br>GAACAATATTTTTGTGCCTAACGCACAAGAAGCTAAAGAGTTTTCTAAAAATATCTATATGAGGAATGTTGAGCAAGAATTCTGGGATAATTACCA<br>AGGACAAAATGTAGTAATATATGATGACTTTGGTCAATTGAAGGATTCTCAATCAACCCCGAATATGGAATTTATGGAATTAATTCGAACAGCAA	Frame 3 5’-3’	ETQ-LVLILK-FNHDINAEDKYFDLYPLNLLKNM-<br>LLGGDYILVAVNLQL-<br>SILCRSSSNATYQRNMNTFNKQPATLKYQASKKI<br>FTGKHDELHVIWEDMIRSAVEDKHVYNSTTSYA<br>FNRAWCCCTRKETATLFGDFSCNYFQLTPYWE<br>GEMNDYTDLNCDPIENTIMALQMSEFEPIWFYLL<br>NKVLERSILEQDYQHQNYCFKYICKIKVSVHWW<br>EVLARSGFFVKLSKCLSVGAMKRLLQKCGDIEL<br>NPGPTYKEICQRKFKRNLHSDKREEWKAQKQIE<br>RQIRLEEEEYVPKNHKKIIKEVEMQMFGTLGTLA<br>SITGNMWLGAKTNNALDKATKSIDTVTTEITKTLD<br>QFRLTFANFSGMMFKSFNILDIISDIIFALLQLSYA<br>KYECKIKSLTVEFVRIMRKYGFGISIDYIRNL<br><br>IMRHRQPSMQIDGKIDFYEYLNPTTIVSLLFVVLS<br>TVFTTVLPKKTHLEEVIKRCGELGRSSKGIKDLN<br>DTLHESVGFMLEYFKVNVLGLTPTEKIEEFISGID<br>RWFDDVRNFLQRKDFA<br><br>DKKSEDIMKDPNVLVEVENIYKRGLEFSREISDK<br>KLQRELTLPFQTHMKYVTDLMKLVDTSGAFGSR	YP_008888535.1 / AGL33499.1, Fex1 / IAPV non-structural polyprotein, 96, 46

contig	nucleotide sequence	RF & →	putative protein sequence	blastp result
	ATATTGCACCTTATCCACTTCACATGGCCCACTTAGAAGACAAACGCAAGACCCGATTTACGAGTAAGATAGTTCTATTAACTTCAAATGTGTTT<br>AACCATTCAGTATCTAGTTTAACTTTCCCTGATGCCTTTAGGCGAAGAATTGACTTATGTGCACGAGTCTATAACAAAGATGAGTACACTAAAGT<br>AGGATATTCTGATGCTGCAAAGAAATCAGTGAAACGACTAGATAAAGAAAAAGTGATGAAAGAAACAGGAAAAATAATTTCAACAGATGTTTATT<br>TGTTGGATATTATGGATCCTGAATCTGGAGCTTTGATTGAAGAAGGTATAACATATGACGAGTTTTTAGAAAAAGCGCAGAAGATGACTAAGAAT<br>GCATTCAAAAGTTCAAAGCTGATGAATGAATTCTTAGAACAGTATGCAGAGACAAGATTCGAACCGGACGCACACAAGGAAGTTAACATGCAAG<br>TACTCCAGCTGAATGATAAATATCAATTTCTTATAGATCAGAGCGTTAGGATGACAGCAGATAATCTTACCCAAGATGATATGCTTAATCCTACG<br>GATGTTCTGAGCTCTTTGACTGACGATAATTATCAGGATGCTATTCACGATCCTCTGGGAGATTTACAGCCTCTTAGTGATCAAGAAATCCAAAT<br>GATTATTGATGGAAATAATGAGTTTGAAGTCTACGACGTTTTAGGACATGTAGTGGATATACAACAATTTATTTCTGATCAATATGATCGATTTTT<br>GAATACAACGTATACTTGTTTTGAAGATTTCCAAAAAATGACAATCCAGGGAGCGCTTTATTTGAAGCGTAATACTATAGTTGAAAAATTACTGAA<br>AGCAAAGAACCATGCAATGACGACGTTGCAAACGTGGGGAGAGAAAGTAAAGAAATTTGTAATGGAACACCCATTCGTTTTCTTTGCTAGTATT<br>TTCTCAGTGTTAAGTGGTATTTTGTGTGTAAGTTATTTTTGGACTAAAGTTTTCCCAAAACCCAATAAGGTAATCCAAACCACATTGATTGATGAC<br>AAAAATGTGAAAATCATTAAGTCAGAAAAGAAATGTGATTTGGAATTGTCGCACCTTACAAAGAAAGAAGCAAATACTGTTTTAAACCATTGTCTT<br>GACAATACGGTATCTTCTGTGAAACTCTCTAACGCTTGGCCAGAGGCAGTTAACATAATTAATAGTTATGCAGCTGCATCGAACAAGCAAATAGA<br>AATAGTAACAGAAAATCCTAATGTTAACCGACATGGCAAACAGTATAAACTGGAAGCGACGTCCAGTGGAGACCCCAATACTCTTAAGCCTAAG<br>AGTATTAAGATCGAAGCAACTACTAGTGGTGACCCCAACACCTTAAAGCCGAAAGTGTTAAAACTTGAAGCTACCACTAGTGGTGACCCAACGA<br>CGACCCGACCCCGTAACATTCGCATTGAGGCAACAAGTTCATCCGATGCTGTAACTTTAAGGCATAATGCTAAGAAGTTTATAGAATCATTCCCT<br>TTAGGAAAGGATGAGATTGACGCTGAAATGCAAATGTGGAAGGACCAAGTAGCTCAAAATCTTATAACGAATAGAATCTTTTCTAATCTCTATAA<br>GATTGGGCGAAAGACAAATGATGAAGTTTTGCCTCTCCTAAATGGACTATTCATCCGAGGAAATATTATGTTAGTTCCAGGTCATCTTCGAGGTT<br>TTTTAAACCAAGAGGATGTAATCGAACTGCGTAGTATTTGGGATGTAGTATTTGAAGTTCCTGTAAAGGATATTAAAATGCTAACAGTAGTCAAC<br>GCTTTAGGAGAAAGCAAAGAAGCGATGTTGCTTGTGTTCCCCCGGTATGTTAGTAGTCATTCTGATTTGGTAAAACATTTCAGCAATGGTGAGA<br>GCATGGGAAAATACAAGCGAGCAGATGTCTGTTTACCGCTTATACGGTATTCAGAGAAGATGAAGAAGTTCTTGATGATGATTTTGGGAAATCA<br>TGAGTGCAGAGCATACGATGAAGAAGTTTTTCTTTGTGATAAGGAAAAAGGAAATTATGTTTTGCGTCAGGGACTCGAATACAAGTGCCCGACT<br>ATTGCTGGTGATTGTGGAGCACCAGTTATTGTGAATGAAACGCAAGTTTTACGAAAAATAGCTGGTATCCATGTTGCTGGTGATAAGGATGGCA<br>TGGCGTATGCCGAGTCGATTACCCAGAAAGATTTAGAAAGAACTTTGAAGAAGATAGATGCGTCACTGCAAATTAAAATAGACCTTGATGGCGT<br>AGTGAAGCATCTCCCCCCCCAAAAAGAAATAGCACTTAATGAAGAGTTTGATACAGATGTCTTGGAATTCTGTGATCTACCAGCTAAGAAATTAG<br>TACCGGTAGGGAGAATACCAGAAGGCTTGCGAGAGCCAGGCAAAACTGAAATCAGACCCAGTTTAATATTTGGCATGATTGATGAAATCAAAAC<br>AAAACCTGCGTATTTGCGTAATGTTTGTGTTGACGGAGAATATGTGAATATTAAACATAAAAATCTTCAAAAGTGTGCTATGGATACCCCCTATAT<br>AGACAAGGACCTTCTTAACCAAGCATACACTTATGTTAAGCCAGTGTGGTTAAGAGGAGCACGGAAAGAACTACAGCGAGTTCTAACTTGGGAA<br>GAAACTGTCATGGGATCAGAAGACAGTGAATACATTTCTTCTATAAACCGTTCAAGTTCTCCTGGTTATCCCTGGATCTTACAGAGACAGGGTG<br>GTACCAAAGGAAAACAAGGATGGTTTGGAGATGAAGAATTTGTATTGAATGAAGAAGTTAGAAAAGCAGTAGAATTTCGTGTAGAACAGGCTAA<br>ACAAGGAATTCGAGTTCCAACCTTGTGGGTGGATACATTAAAGGATGAAAGACGACCAATAGAAAAAGTTGATGCGCTTAAAACACGCGTCTTT<br>TCTAATGGTCCGATGGACTTTTCATTGGCTTTTAGACAATATTATTTGGGCTTTATAGCTCATTTAATGGAGAATAGAATAACCAATGAAGTGTCG<br>ATAGGCACTAATGTTTA		PRTQPIVIWLYGESGVGKSGMSWPLAVDLNNIF<br>VPNAQEAKEFSKNIYMRNVE<br><br>QEFWDNYQGQNVVIYDDFGQLKDSQSTPNMEF<br>MELIRTANIAPYPLHMAHLEDKRKTRFTSKIVLLT<br>SNVFNHSVSSLTFPDAFRRRIDLCARVYNKDEY<br>TKVGYSDAAKKSVKRLDKEK<br><br>VMKETGKIISTDVYLLDIMDPESGALIEEGITYDEF<br>LEKAQKMTKNAFKSSKLMNEFLEQYAETRFEPD<br>AHKEVNMQVLQLNDKYQFLIDQSVRMTADNLTQ<br>DDMLNPTDVLSSLTDDNY<br><br>QDAIHDPLGDLQPLSDQEIQMIIDGNNEFEVYDV<br>LGHVVDIQQFISDQYDRFLNTTYTCFEDFQKMTI<br>QGALYLKRNTIVEKLLKAKNHAMTTLQTWGEKV<br>KKFVMEHPFVFFASIFSVL<br><br>SGILCVSYFWTKVFPKPNKVIQTTLIDDKNVKIIKS<br>EKKCDLELSHLTKKEANTVLNHCLDNTVSSVKLS<br>NAWPEAVNIINSYAAASNKQIEIVTENPNVNRHG<br>KQYKLEATSSGDPNTLKPKSIKIEATTSGDPNTL<br>KPKVLKLEATTSGDPTTTRPRNIRIEATSSSDAVT<br>LRHNAKKFIESFPLGKDEIDAEMQMWKDQVAQN<br>LITNRIFSNLYKIGRKTNDEVLPLLNGLFIRGNI<br><br>MLVPGHLRGFLNQEDVIELRSIWDVVFEVPVKDI<br>KMLTVVNALGESKEAMLLVFPRYVSSHSDLVKH<br>FSNGESMGKYKRADVCLPLIRYSEKMKKFLMMI<br>LGNHECRAYDEEVFLCDKEK<br><br>GNYVLRQGLEYKCPTIAGDCGAPVIVNETQVLR<br>KIAGIHVAGDKDGMAYAESITQKDLERTLKKIDAS<br>LQIKIDLDGVVKHLPPQKEIALNEEFDTDVLEFCD<br>LPAKKLVPVGRIPEGLR<br><br>EPGKTEIRPSLIFGMIDEIKTKPAYLRNVCVDGEY<br>VNIKHKNLQKCAMDTPYIDKDLLNQAYTYVKPV<br>WLRGARKELQRVLTWEETVMGSEDSEYISSINR<br>SSSPGYPWILQRQGGTKGK<br><br>QGWFGDEEFVLNEEVRKAVEFRVEQAKQGIRV<br>PTLWVDTLKDERRPIEKVDALKTRVFSNGPMDF<br>SLAFRQYYLGFIAHLMENRITNEVSIGTNV	
TR30079| <br>c2	GTTCGTTCCAATAGACACTTCATTTGTAATTCGATTTTCCATCAGATGAGCTATAAAGCCCAAATAATACATTCGAAAAGCAATTGAGAAGTCCAT<br>TGGTCCATTTGAGAACACTCGTGTTTTCAACTGATTAACTTTTTCAATAGGTCGACGTTCATCTTTCAATGTATCGACCCACATCACAGGTAATCG<br>TTTTCCTTCACGAGCTGCTTGTAGTCGTCGTTGTACGGCTAATTCGACATCTTCATTAAGAATAAATTCTCCGTCAATGCCAAACCAACCTTGCT<br>TTCCTTTAGTTCCTTTCACTCTATCCTTGATCCAAGGATATCCTGGGGAACTACTCCTATTAATTGAAGAAATATACTCACTTACATCAGAGCCAC<br>AAATGGCTTCCTCATAAGTGAGTACTTTCTTCAATTCATCTCGCATACCTTTTAACCAAACAGATTTGGTTAACTGATATGCTTCCTCGATCATGT<br>CCTTGTCAATATGAGGTGTATCCATAGCACATTTCATAAGATTTCTGTGTTTCATATTTACAACTTCACCATCTACTCTTACATTACGTAAGTAAGC<br>TGGTTTAGTTTTAATATCAGAAATCATTCCATGTACTAAAGAAGGCCTAATATCAGTTTTGCCTGGTTCGAATAGGGGTTCGGGAAGTCGACCTA<br>CTGGTATCATCTTAAGAGCTGGAAGATCACAAAAAGTGAGAGCTTCTGGCCCAAATTCAACACCAGCAGGGATTTTGGGTTCGGTTCTAGAAAA<br>ATCTAATGAAGAATCTAAGTCCAATTGGATTTGCATCGTAACATCTATTTTACTAAATGTACGCTCCAAATCTTTCTGGGTTATAGACTCTGCATA<br>AGCTCGGCCACATGCTGCACCAGCAACATGTATACCAGCTATCTTTCTAGTAACCTGTGTTTCATTGATGATTAGTGGAGCTCCACAATCACCAT<br>CAATTGTCGGCATCGTATATTCTAATCCTTGTCGTAATATATATTGTCCTTTCTTAGCATCGTTTAACGTATAAGTTTTATCACATGCCTCTACTTT	Frame 2 3'-5'	VFSNGPMDFSLAFRQYYLGFIAHLMENRITNEVS<br>IGTNVYSQDWRKTAKKLLAKGKKVIAGDFSTFD<br>GSLNSGIMALFADLANEFYDDGAENALVRHVLL<br>QDVYNSMHICGDSVYGMTHSQPSGNPATTPLN<br>CFVNSMTMRMVFVLCAEQAHQKMTMKDFDRH<br>VSMVSYGDDNVVNFSDEVCGWFNMDTITAAYA<br>RLGFTYTDEAKSTNGTVPIWRDISQVAYLKRNFR<br>YDDNRGVWEAPLAMDTILEMPNWCRGGLDILE<br>GTKCNAENAIMELSMHEESVFDFWSKKIANAFY<br>AKTGECLAINTYRGYAQDRYLQYYLN-<br>FLWLPSS-ENVF-LFSQGLEIRVEGPI-	AHL83499.1,<br>KBV non-<br>structural<br>polyprotein, 89,<br>94

contig	nucleotide sequence	RF & →	putative protein sequence	blastp result
	GTCACACTCAATAAGAGAAGACATAAATCTTTCTAATTTTTGAGAGTAACGCAGTACAGGTAATGTGACCTCGCATCGCTTAAACTTTGACATTG<br>ATTCAGCAGTTTGGAAATGCTTCACAATGTCAGAGTGTTGGCATACGAACTTAGGGAACGCTAATAATGCTGCTTCCTTGGATTCACCAAGAGC<br>ATTCTCAACTGTTATCTTCTTAACTTCTTTCCATGGTACTCTAAATACCACATCAAAAAGATTTCTAATTTCAATTGTATCATGTTCTGCAATGAAT<br>CCAGTCAAATGTCCGGGTACTAACATCACATTTGAACGAATAAATAATCCATTCAAAAGTGGTGTTACTGTGTCGTCGTCGTTTACTAAACAAAT<br>TTTATACAGATTTGTTAAAATTCGGTTAGTGATCAATTTCTGCGCCACCTGGTCTCTCCACATTTGCATAGAAACATCAACTGTATCATATCCAGT<br>TCCTTCAACATATTTTGTTGTTGGTCTTTTCAATGTTACAGCATCAGAACTTGCAAATGCTTCAATCATTCGTTGTGTGTTTTTAGCTAAAGTAACA<br>CAATCAGCAGATGTCTGAGCTTCTATAACAACTGGTCGTGGTTGTTTAAGAGTCATATTATCTCCTGATGTTTGTGCTTCAATAACTATCGGGGT<br>CCTGTTTTTGTAAGTAGCCAAATCAGCAGAAGCAAAAGCTTCTACTTTTGGTGTATTTACAAGCGATTCCGGGTCTTCTTCAAAGAAACGATTCA<br>TCTCACCACAAACCAATTCAATATATTTATTTTCATAAGTAAAGAATCGGTTCTTGGTAATTAAAATCGTTTTATCAAAGAGTTTCGAGTGGTTATC<br>AATAATACTCAAAATGAATTTAGTCACTTTGGGTACAATGACAACACGATGTCTGGGTTTGAGCAAGGCTGTCAAATGATCTTCAACATCTCCAA<br>CACGCATGTTGGATACGTCTAATGTTTCATCTAACTGCCAAAACTTATTGAGGTCTCGGTGTGGTATGACAAGAGTCACACCAGTATTAACAAAA<br>TGTCGTTTTGTAGCTTGTGGTTTCTTCTTTTCTCCACTGCAAAGCCATTTCCAAAATCCTACTACGGTAAGAATACCTAATAGGGTTCCTAAAATG<br>GCCATTGCTGTGGTCCAAGGGTGTTCCTGCACATATTTGACAGTTTCTTCAATCCAAGTCTTGCAAATCGCAAGACCTGCATCCAAAACTTTCTT<br>CAAATATGATACGCCACTTGCGACTTTCTGGTAATACTTCATTTCCTTAATTCGTTGCATTTGTTCGTCATAGTCTAATGGAGCAAGATCAAAAGC<br>GAGTTTTGATATCTTTACCACTTGTTGTTTACAATCATAAACAACACCTGTGCATGAATCAATCATCTCTCGCAATCTATCTTGTTCAATGGGAAC<br>AAGGTCATCATTAGGAGCATCGATTTGCATCTGTACTTCTAATGTATCCTTGAACTCATCATCGTCGTATTCGGGTTCAGGATGGATAAAAGTAT<br>CTCTACTTCTATCTTTACGTTGTTCGGCATAATTCATAAGAAAATCATTGAGTTTGGCAGATTCATCTCGACACGTCTTGGTTTTCTCCAAGCACA<br>TGTCTAAAAAGTCTTCATACTCAATGTCAGTCTTATATTTTTCTCCGGTCTCTGCATTCACTAAGTCAACCAAATATGGTTTCGTTGAGTGAATAT<br>CTCCTGTCAACTTCTGCACCTTTTCACGATCTAATCGTTTAACCATGCATCCTTTTTGCTTTGAATAACCTTCCTTCGTATACTCTTCCTTATTGCG<br>TACTTCAGCACACAGATCTACTCTGCGTCTGAAAGCATCGGGGAAAGTAAGCGAATTTACAGATTGTTCAAAAACATTGGATGTCATAATTATTA<br>CTTTCGATGTGAACTTCGTCTTTCTCTTATCTTCTAAATGGGCCATGTGTAGGGGGTAGGGAGCGATATTAGCGGTACGTATCAATTCCATAAAT<br>TCAGGGTTTGGATTAGCTGTTGAATCTCTCATTTGTCCAAAATCATCATAAACTACAATATTCTGTCCTTGATAGTTATCCCAAAATTCCTGTTCTA<br>CATTTCTCATATAGATATTCTTGGAAAAGTTACGCATCTCCGTTACATCATCTAATAAACTATTATTTAAATCTACTGCTAGGGGCCATGTCATAC<br>CTGACTTACCACGTCCACTTTCACCATATAGCCAAATGACTACAGGTTGAGTTCGTGGTTTGTTTCCAAAAGCACCAGAGGTATCTACTGCTTTG<br>AAAACTTCAGTCAAGTATCTCATGTGGGTATTAAAACTAATTGTTAATTTTGATGGGAGTTTCATATCAGAAATATTGCGGGCAATGTCCAATCCT<br>TGCTTGTAGAGGTTCTCTACACGCTGAATTTCATGAACATCCTTCATTATTTCTTCAACAATACTTTTTCCATCAAAATCTCCATCTAACTTAATCT<br>TATGTCCTACTAGAGCTCTTACTTCATCGCACCAAGCTTTATAGCCTTGTACTAAAAGTTGAAGCTCTGCTTCTTGTTTTAAACCTAAAGTATGAA<br>CACCAAAATGTTCCAACATAGTTGAAATAGAATTATTCAAAACAGAATTGAGATCAGAGACGCCTTTTGCAGCACGTCCTAGATCTCCAATTCTC<br>TTTATCATTGCTTCCATTCCAGTCTTTGTCGGAAGAGTTCTGGTGAAAATAACACTTAATACTAAAAATATAGCGCTAACAATAACATTGGGTTGT<br>AAAAGAGTTGTGTAATCTGGCATATCAATTTGCATTTTAACTTGAATAATGGGATTATCCGTGTCTAACGCTTGTCTACCAAATGTTAACAACTCC<br>TTTATTTTATCAATGTTAATCTTAACGCTACCTACATACTTCTTAATTAATCTAAATACTTCTACTGCTAAAGAAGCTAACTTCATTGAAGCTTTCGC<br>AAAACTTACCTGTAACAAAGCACTAATGATATCTATACAGACATCAATAATACTAACAGTTCCAAATAATTTTTGTTCACACTTCGCATAAGTCCTT<br>CTAAAGCCTGCTAACGTTTCTTCTAATTGTGGTATAAGGTTGTCCATTATGTGATTAGATTTATCCAGAGTGTCATTGAATTTATATGCAGTTGAC<br>TTAATAATATCCTTTTCTTCTTGGAAATTAAAAAGTCCCTGCATAGTAACATCAATAACATCTTTCAATTTCTTGGGTTTTGTCTGGTGGGAACGAA<br>ATTCTTCTCTTATTACTTTATCAATGTGTTGTTGCATTTTGATTTCTTCATAAGATTTAGAAACTCGTTTGCGTTGTCGTCGTCGTTGGCATTGTTC<br>TTTGTAAGAAATATCAGTGGGTCCTGGGTTCAATTCAATATCTCCGGCTAAGAGATTAAGAATTGATTCCCAAGTTCCACATTTATATAAATTATT<br>CAAAAATCCAATGCGATATAATTCATACCAAAAATGTTTTGATACATAGATGGTAGGAAATTCTGGCATTCCTCTCCAATTCATTGGTACTGGTAC<br>GAAATCAAGATATTTAAGCAATAATGTAAACCAAACTTGCTCAAATTCGGCTGACATTAATGCATCATATAAATTTTCATGATATTCGCAATTTAG<br>GTCCCAATAAGTTGTGTCCTTCGTTCGTGTATCTTCATCATAAATTGATAGAGTAAACCCTATACAATTTATGTCTTCGTGTAACATGTGAAAAGC<br>GTATTGGTCAATATAAACCATCTTTAAAATTTGTTGGCGATGTAAATCATCAAGTGAAAAGAATTCACTTGGTGGCATACTAATCACCTTCATACT<br>TGAAAGATAGGTTAAAGTTGCTGGTTGTTTTGTCATCTTGTTATTTTGTTGAGTTGAGGAAAACATGTTTTGGCGAAATCGTCCGGCTCCATTAC<br>CACTGCTCCGACACAGGAATAAAAAGATTACAAGTTAACTGTGACCTTACTGTGATATCCACTGAGACTCACAGATACTTTATATAGAAATATAG<br>AGGTTAATAATCGAAATACTTGTCTTCAGCATTATTAACATATTTAAATTCTATTAAGTATAGGTACTAATCTTTTTAAATTCAAACTCCTAAAAGGA<br>GGAATTCTCAGGTAATGTATTTATCAGAATTTAAAGCCTCACGTCTTTAAATAGAGCTATCTCGTCCCTCATTCTTTTCACGGAAATATTGGGAGA<br>TTACTAGACTTCATCTAAGATAGTTATTTAAAGGTTCGTAATCCGGTTGTGTTTATGTAGTTTAGACAGTTACATAAGCTGTTTGTCGTTATAATCA<br>AATCATAGAATTAAAGGCTAAAATGGTTTCGTCGACTAAAATTCGTGTTCGTTATAAGTTGCTGAATTATTCTACAGCTCGAGTCGGTAGCTTATA<br>TTCTCCACTCCATACGGGGGAATGACGCCCCTCTGGTTACTACCGAGCGCCACATGTGAGTTCATTATCGCATGAACAAAGCGTCAAAATTGTA<br>CGTAGGGAATAAAGTCCA		DEVLRWQPHQNL-<br>RLGAAISGCYSSVKFIWRVPKHQTC-<br>YSNELRTEFYPYKYRT-HDNGRKQC-<br>NGPNHNLS-CGNSKSVHYPLGAGHCSNWQ	
TR31304| <br>c0	AAAGCCCCGTATGCTTGGAGACCAGGTTTGTTTGCCGACTTACGGAATGTCGTTAAACTCTAGGCTTTCCGGATGGCTTCTAATTATCCGAGGC<br>CCGTACGTGGGCTCGAAGTTTAGTAGAGAGTACGCGTACAAAAGTCCTAGTTGTTTTCATACATCAAGTGGGTTAAAAGCCCCGTGTACTTGGA<br>GGCTAGGCAACACGATTACAGTAGCCTGTACTACCTGAAGCATGTAGTAAAACTGCACAGTTGAACATTGTCACAAACGGGCTGATAGCGTATA<br>GCTGGATTGTTGAGAGATAGCATCTTGCGGGATGTGAAAGAGCATGTTAGTCATGTATTATGGCTTTTCTCAATACGCAGTCTTTGCTATGCGT<br>GTGTAGGCAAAAGGAAGTGAGAGAGTTGACCGTCTCTCATGGGTATCTAGGTTATTCAGAGTACGCTGCTACTGCGGTAGCGGACACAACCCA<br>GGTGTTCGGGTCTCTGTCTTAGAAACTATATGAAAGGCGAAAGTTGTGATTGAGCTTTCTAACGTCACTCTTAAATGGTACGTGTCGATTGAGTT	Frame 3 3'-5'	SPVCLETRFVCRLTECR-TLGFPDGF-<br>LSEARTWARSLVESTRTKVLVVFIHQVG-<br>KPRVLGG-ATRLQ-<br>PVLPEACSKTAQLNIVTNGLIAYSWIVER-<br>HLAGCERAC-<br>SCIMAFLNTQSLLCVCRQKEVRELTVSHGYLGY	ABS82427.1,<br>BQCV non-<br>structural<br>polyprotein, 85,<br>97

contig	nucleotide sequence	RF & →	putative protein sequence	blastp result
	CATAGCAATGAATAGCCAACCCTATAATGATGGAACCAATTTACGAAAATAAACCTAATGTATTTTACTATCCTTTCAGACACAACTGGGAACCAA<br>GAATCTTGGTTCTTACTGATAAAGAAGGGCGAGAGAGCGTAAACAAAAAATTCTTAAGGAAGTTCTCTAAACTTAGGAAATTATGTCTACGCTTA<br>CAAAAAGATACAACGTTTTGGAAGAATACATCTGGTAGGCTGGACATGATCCAGAAAGATATTGAAAGTATGGTTGCACAGAGTGGCCCTGTTA<br>TGAGTCAAAGCTTAAGTGACAGGGTTGACCGAATGTACCAGGAACTGATACAGACTAACGTAGCCCAGATGAATTTTTCTATACCGTTTTCGCA<br>CAATTTGTCCAAGGATTCGAAGGCGTTTCTTACTCAAACCGTAGATACTTCCATTGGTAGCATGAGGCAAATTTCTAATGAAACAGTAAATACTA<br>TCAAGGAAGTGTTTGAGACATTTTTGACCAAGATGACAACTACTTTTGAACAGTGTGCCGAGGCTTTTAATTTTACCATGAGTTTTATGAGATTTC<br>TAGCACTAGCCTATGCTATTTTCTTTCTCATATCTCGAGCGACGAAATTTTTCGGAGCTTCTTTTTCGACTATTTCTACTGTCTTGATAACTCTTAT<br>CACTATGACAGTCACGGATAAGCTGGTTAAGAAGTTCCTTGTGGACTATTACTCGAAACATATGGAGAATGCAAAGATGTTGGCACAAGCAGGA<br>GAAGAAGATTCGTCATTTCTGTCTTATGTTATCCCACCATTGTTCCTTGAATCTGTAGTCGATTCTAAATCAAATTTATTCAAACGAATTTGGAGA<br>AGTAAAGACTTGGATTTAATAGTTAAACGGTTATCTTATTTTGGAGATTCCAAGATATATAATGCGTTTGATAATATTGGAACGTGGTTAACAACT<br>ATGATAAATAAGACAGTCAATTATGTTAAGGTGACGCTTTTAGGTCATGATCCCGAAGAGTTTTTAGAAAATCAGAGTGATCCGTTGGTAGATTG<br>GGAAGAGCAGTGTCGTAAACACTTTGTGTCCGACACTGACAGAATAGCAATATATACAGAGTCCACATTGGCGGATATTAAGCGACTGTACAAT<br>ATGGGTGTTAGCTATATGCGACACCCACTCTATAAACCTCATCAACGAATTATCTCTGAGATCTTAAATCAGTTACTCAGATTTGCCGACAAAAT<br>AAAGAAAAAGGTGGGCACGGATGCCTCTGTCCGAAACCCACCAGTTACTCTATACTTGTATGGAGAGACAGGAGTTGGTAAATCTACTCTCACA<br>TATCCATTGTGTGCTACCCTTCTCAAAGCTATCTTTACGAAGGAAGGAAACACAGTGATGCTTGATTCCCTTAAACAACATTATAAGGAAATGAT<br>ATATGTGAGAGCTGCCGAACAAGAGTTTTGGGATGGCTATACACAACAACTAGTCACCGTCTTTGATGATTTCAATCAGCAAGTTGATTCCTCA<br>GCTAATCCTAGTTTGGAGTTGTTTGAAATTATCAGGAGTTCCAACATCTTTCCATATCCACTACACATGGCGTCGATTGAGGAGAAGGCAAACAC<br>TGTTTTCCAATCTAAAGTAATATTGTGTTCATCCAATAATAAGACTCCTAAAACTGAATCTTTGAATTATCCGAAGGCATTATTGAGAAGATTCGC<br>GAAATTTGTTGAGGTGAAGAGGGCTCCTTCCGAAAATGGTACATTTTCTACTGATTGTTACACTTTCGTGCAATACGATCCGTTCGATCATTGTA<br>ACATTGTCAAATCAATGTCTTTCAATGAATTGATAGATGAAGTAGTTGCGATGTACTTCCAAGAAGGAGAATTTGTCTCTTCTGTAGACAAATTTA<br>TTATGGAGAACGTGTTTGCTCAAGGTGGGGGTTTATCATCTGACGAAGATGATAATTTTGTTGAGGCAAGTTCCTCCAATGATAGTGACTTGGA<br>TGAAATCTTGACACGCGAGGAGAAGAAAGAGATCGTGGAATGCATTAATGACCAGCTGAAGAATGAGAATGACACTAAAACCTCTCTCCAATTA<br>ATAAGAGACTATTTCCATTGCGCCAAGGAATCGCTACAGGAGAAATTTTTAGCTTATCGTCAAAAGTATTCTTTTGATGAATGGTTTAATTTCTCC<br>AAAGCATCTAAAGTGGTTTTAGGAATTTTGTCTCTGGTGTTGGTTGGATACGGCATATATTCCTATGTCAAAGGAACATCCGTTAAGGACTCGGC<br>TGAATCTTACGAACCAAAAGTGGCGAGTAAGAATAAGGTCGAAGGTAGTGACGGTGCTCCTGAGGCTTACGAACCTAAGATCAGTAGAAAAGT<br>AAAAGTTGAGGGATATAATGCTAGAGAGATTAAGCGGAAGGTGGAAGCTTATGCTCCTAAAGTAAATAGAGCTGTCCGTGTAGAATCTAACGAA<br>GTCATAATTCATCCCAATTCTTTGGTAGAGAAGGGAGATGATATTGTGCAATCTGAGGCATGTAGCGATATAAATGCGTCAGAACAGCTTACGG<br>CAGTAACTACAAATAATACTTATGTTATGACCATTTTATCTAAAGAATCAGTCATTAGAGTTGGACACTGCATCTTTCTGAAAGGAAAGATTGCAG<br>TTGCCCCCGGTCATTACTTACGTATACTTCAGAGAGCATATGAATTGGATGAAGGAGCAATTCTCCAATTCAATCATCCCTACGGCAAAAGAAAT<br>TTCTTTACGTTTATAGGTGACATCCAGTTCTCTCTATACAAGACCAAAAATATATCTAAAACAAATGATTTGGATTCCCGTGACTTGATGCATTTC<br>GTTGTAGATAAGTCAGTAGTTCACAGGGATATTTCTTCATTTTTCTGTGAACGTTTAGAATTACAATCTGTAGGTTCGACTCGAATCCAATTACCC<br>GTCATGCGATGGGTAAGAGATGTTGGATATATTTTCGTGAAATCGGGACAAGGTACCAGTTGTATTAAAAACGTTTCTAACGTTAGCTATATGAC<br>TGATACTGATCCTGATGCTCGAGAAATCCGATTGAGAGAAGCGTGGGAGTATTCTTTAGAAACGATATCCGGTGATTGTGGGGCTCCTCTTTTT<br>GTAACCAATAGTAAAATAGGACCTGGTAAGATCATAGGAATTCATACAGCTGGAGGTCATCGTTTTGGAGTTGGCAGTTGCTTTGCCACTCCAT<br>TTTATATAGAGGATGTGAAAGAGATCTTGTCTCGGTACGACTATAAGGCGCAATCTTGTGCCTTTGAGTTGGTGCTGCGACAGGACCTCGAACC<br>ATGTGCTCTACCTAACCATTTGGACGAATGTGAGTTCATCTTAATGGGAAAGGTTAAAGATCCTCCAGTTCAACCATCTCGAAGTAAAATTTCTC<br>CATCTCCGTTATATGGACACATAACTGAACCCTCTAGTGCCCCTACTTGGTTATATCCCAGAGCTTGATGGACAGATTTTTGACCCTCTGAAGTA<br>TAGGACTGCTAG		SEYAATAVADTTQVFGSLS-KLYERRKL-<br>LSFLTSLLNGTCRLSS-Q-<br>IANPIMMEPIYENKPNVFYYPFRHNWEPRILVLT<br>DKEGRESVNKKFLRKFSKLRKLCLRLQKDTTFW<br>KNTSGRLDMIQKDIESMVAQSGPVMSQSLSDRV<br>DRMYQELIQTNVAQMNFSIPFSHNLSKDSKAFLT<br>QTVDTSIGSMRQISNETVNTIKEVFETFLTKMTTT<br>FEQCAEAFNFTMSFMRFLALAYAIFFLISRATKFF<br>GASFSTISTVLITLITMTVTDKLVKKFLVDYYSKH<br>MENAKMLAQAGEEDSSFLSYVIPPLFLESVVDS<br>KSNLFKRIWRSKDLDLIVKRLSYFGDSKIYNAFD<br>NIGTWLTTMINKTVNYVKVTLLGHDPEEFLENQS<br>DPLVDWEEQCRKHFVSDTDRIAIYTESTLADIKR<br>LYNMGVSYMRHPLYKPHQRIISEILNQLLRFADKI<br>KKKVGTDASVRNPPVTLYLYGETGVGKSTLTYP<br>LCATLLKAIFTKEGNTVMLDSLKQHYKEMIYVRA<br>AEQEFWDGYTQQLVTVFDDFNQQVDSSANPSL<br>ELFEIIRSSNIFPYPLHMASIEEKANTVFQSKVILC<br>SSNNKTPKTESLNYPKALLRRFAKFVEVKRAPS<br>ENGTFSTDCYTFVQYDPFDHCNIVKSMSFNELID<br>EVVAMYFQEGEFVSSVDKFIMENVFAQGGGLSS<br>DEDDNFVEASSSNDSDLDEILTREEKKEIVECIN<br>DQLKNENDTKTSLQLIRDYFHCAKESLQEKFLAY<br>RQKYSFDEWFNFSKASKVVLGILSLVLVGYGIYS<br>YVKGTSVKDSAESYEPKVASKNKVEGSDGAPE<br>AYEPKISRKVKVEGYNAREIKRKVEAYAPKVNRA<br>VRVESNEVIIHPNSLVEKGDDIVQSEACSDINASE<br>QLTAVTTNNTYVMTILSKESVIRVGHCIFLKGKIA<br>VAPGHYLRILQRAYELDEGAILQFNHPYGKRNFF<br>TFIGDIQFSLYKTKNISKTNDLDSRDLMHFVVDKS<br>VVHRDISSFFCERLELQSVGSTRIQLPVMRWVR<br>DVGYIFVKSGQGTSCIKNVSNVSYMTDTDPDAR<br>EIRLREAWEYSLETISGDCGAPLFVTNSKIGPGKI<br>IGIHTAGGHRFGVGSCF<br><br>ATPFYIEDVKEILSRYDYKAQSCAFELVLRQDLE<br>PCALPNHLDECEFILMGKVKDPPVQPSRSKISPS<br>PLYGHITEPSSAPTWLYPRA-WTDF-PSEV-DC-	
TR86646| <br>c0	AATAAGAGATTCTGGAAAATTTGTAGTATACAACTGCGTCTCAGAAGGAGTGGCAGAGGACCAGAGTCCGGTTTGAATTATAATAGGTCGAGCC<br>AGAAAATCTTTGATCGTGTGAATACGGTCTTCACGCGTAGTCATTGACAAATAATCAGTCGATAAATTAACAATGTCAGGGAGCGCGCTAGTTG<br>ACGGTGTAACTCCTTCACTAGTAAAGTGAACGATTTCTCGTTGTTCAGAGGCGATCTTCCGATCTTCATTCTCGATTTTTGTGTTTTGGTTTTCTT<br>GAAAGTTAGCAGGTAAATTTCTTTCATATTACGACTACCTAATCATTAATACGGCAAAGAGGGTACCCTGGATATTGTGGGGCTGCCACTAGGC<br>ATCCTGGGCGGTAAAGTTAAATAACTAACCTAGATATTAAAATAGCACTACATCATTCTTGATTAACCTCTTAAATTTATAAAATAATGCAAGATCA<br>CATTTTAACCTAAAATTCATAAACAGCATCTGCAAGATATTCAATATCGTGGAG	Frame 3 3’-5’	PRY-ISCRCCL-ILG-NVILHYFINLRG-SRMM-<br>CYFNI-VSYLTLPPRMPSGSPTISRVPSLPY--<br>LGSRNMKEIYLLTFKKTKTQKSRMKIGRSPLNNE<br>KSFTLLVKELHRQLARSLTLLIYRLIICQ-<br>LRVKTVFTRSKIFWLDLL-<br>FKPDSGPLPLLLRRSCILQIFQNLL	no conserved domains
TR94176| <br>c0	GTTACTGAGAAGGGTGTGGATTACGTCCGAGCTACTATAAGTCCTCAACAGACTTATGGTAGTGACGTCGCTCCAACTACCCATATCAGTACTC<br>CTTTGGCAATAGAACAAATACCAATAAAGGGAGTCGCAGAaGTTCCAAATACCGTACTATGCTCCATGTTTGTCATCTTCGTTTAGAGCGAATTC<br>AGAAACATTTTACTATAGTTCAGGTCGGAATAATCTCGATATAGCCACTTCACCTCCTTCCATTAATCGCTATTATGCGGTTGGTGCGGGAGATG<br>ACATGGACTTTTCCATCTTTATCGGTACGCCGCCCTGTATTCATGCATCACAGACAGCTCAATTTACCAAGATAAAACAAGGTAAAGTGTATGAC<br>TTGAGGTATGATCAGTATGACCCTTTTAGGGAAGTCCAGGACGGTACGGCGTTCCTCAATGCTCGTAGTATTGAGGATAGCGATTTGTTGTGAG<br>CTCCTTTAGAGGGAGGGCTCACTTTATCTATTGCTTAAATCGGTAAGCCACAAATTTTTCTAAGTGTCATGAGTTTCTTCTCGGTTCTTCTCATGA<br>TTACTAATCGAACCGTGTGTAGAGTCAGAATGTTGTGGTTTACGTTTCTT	Frame 1 5’-3’	VTEKGVDYVRATISPQQTYGSDVAPTTHISTPLAI<br>EQIPIKGVAEFQIPYYAPCLSSSFRANSETFYYSS<br>GRNNLDIATSPPSINRYYAVGAGDDMDFSIFIGT<br>PPCIHASQTAQFTKIKQGKVYDLRYDQYDPFRE<br>VQDGTAFLNARSIEDSDLL-APLEGGLTLSIA-<br>IGKPQIFLSVMSFFSVLLMITNRTVCRVRMLWFT<br>FL	AFQ89856.1, BQCV capsid protein, 99, 98

Supplementary methods

Primer design and sequencing of extended contigs

Primers (Supplementary Table 2) were designed using NCBI's Primer-BLAST (Ye *et al.* 2012). The organism in the Primer-BLAST pair specificity settings was Hymenoptera (taxis: 7399) and the maximum product size was 1000 bases. All other settings were the defaults. For the *Linepithema humile virus* 1 (LHUV-1) genome discovery the IVA extended LHUV-1 contig was used as the template. The IVA extended n1905 contig was used as the template for the 5' end of the genome and the Kashmir bee virus (KBV) genome (NCBI GenBank accession AY275710.1) as the template for the 3' end. For LHUV-1 genome extension, cDNA from Argentine ants previously found to have a high viral titer of LHUV-1 were used. The same was done for n1905 genome extension but with Argentine ants with high loads of replicating KBV. The PCR reactions contained 2 µL of template DNA, 12.5 µL of 2x MyTaq™ Red Mix (Bioline), 8.5 µL molecular grade water and 1 µL of each the forward and reverse primers (10 µM) for a total volume of 25 µL. The PCR thermal cycling conditions consisted of an initial denaturation at 95°C for 120 s, followed by 40 cycles of denaturation at 95°C for 20 s, annealing temperature was 57°C, 60°C, 63°C depending on the primers used (Supplementary Table 2) for 10 s and 72°C for 30 s, with a final elongation at 72°C for 5 min. The PCR products were visualized with agarose gel (1.5%) electrophoresis. PCR products were sent to Massey Genome Services (Massey University, Palmerston North) for both forward and reverse Sanger sequencing.

Supplementary Table S2: Primers designed to recover extended viral contigs using Sanger sequencing.

Target virus	Primer name	Sense	Sequence 5' → 3'	Product length (bases)	Annealing temp. (°C)
LHUV-1					
	6665F	F	AGGACTTAGCACGACAACGG		
	7295R	R	GGGGCCCATGAGTTCTGTAAG	631	63
	6151F	F	TGATAATCGCGGTGTTTGGG		
	6777R	R	CTGCAGTGTCCTGCACCAAG	627	63
	5725F	F	AGCAGAGAACGCATTAGTGAGA		
	6260R	R	CGGCATTACATTTGGTTCCCT	536	60
	5134F	F	GTGGTTAAGAGGAGCACGGAA		
	5853R	R	TTAAGTGGTGTCGTGGCTGG	720	63
	4711F	F	GGCGTATGCCGAGTCGATTA		
	5286R	R	CCTTTGGTACCACCCTGTCTC	576	63
	3960F	F	GACCCCGTAACATTCGCATT		
	4811R	R	GCTTCACTACGCCATCAAGG	852	63
	3416F	F	ACGTGGGGAGAGAAAGTAAAGA		
	4103R	R	GAGCTACTTGGTCCTTCCACA	688	57
	3040F	F	TCAGAGCGTTAGGATGACAGC		
	3575R	R	TGTCATCAATCAATGTGGTTTGGA	536	60
	2573F	F	CGCAAGACCCGATTTACGAG		
	3170R	R	GAGGCTGTAAATCTCCCAGAGG	598	63
	2172F	F	CCCTACCATTCCAGACTCACA		
	2686R	R	TGCACATAAGTCAATTCTTCGCC	515	63
	1695F	F	TGCGACATAGACAACCAAGCA		
	2328R	R	GCAAGTGGCCAACTCATTCC	634	63
	1175F	F	GAACTCAATCCCGGACCCAC		
	1874R	R	AAGAGCGTCCTAACTCCCCA	700	63
	793F	F	AGCATGGTGTTGCTGTACCC		
	1380R	R	GCAAGAGTGCCTATGGTTCC	588	63
	359F	F	ATCTTTGAGGGCCGTTTTGG		
	920R	R	TTGGGTCACAATTTAGGTCCG	562	63
	6F	F	ACCCGCTAATATATCGGATTCTAGT		
	595R	R	ACACAGGATGCTCTACAACTGC	590	60
n1905-KBV	10F	F	CCCTACGTACAATTTTGACGCTT		
	365R	R	AGCCTCACGTCTTTAAATAGAGC	356	57
	251F	F	AACACAACCGGATTACGAACC		
	796R	R	ACATGTGAAAAGCGTATTGGTC	546	57
	579F	F	GTGTCGGAGCAGTGGTAATG		
	1290R	R	CTTGGGTTTTGTCTGGTGGG	712	63
	1133F	F	AATTGAACCCAGGACCCACT		
	2022R	R	AGCTCTTACTTCATCGCACCAA	890	60
	1854F	F	AGATCTAGGACGTGCTGCAAA		
	2802R	R	TCCTGTCAACTTCTGCACCT	949	60
	2653F	F	TCGCTTACTTTCCCCGATGC		
	3156R	R	ACAATCATAAACAACACCTGTGC	504	57
	3006F	F	CCATCCTGAACCCGAATACGA		
	3520R	R	TGAGGTCTCGGTGTGGTATG	515	63
	3344F	F	ATGTGCAGGAACACCCTTGG		
	3795R	R	AAGCGATTCCGGGTCTTCTTC	452	63
	3560F	F	ACATGCGTGTTGGAGATGTTG		
	4389R	R	GCATACGAACTTAGGGAACGC	830	57
	4152F	F	AGTAAACGACGACGACACAGT		
	4931R	R	AATAGGGGTTCGGGAAGTCG	780	60
	4704F	F	ATGTGGCCGAGCTTATGCAG		
	5200R	R	AGCCACAAATGGCTTCCTCA	497	57
	4911F	F	TCGACTTCCCGAACCCCTAT		
	5839R	R	GGTTTCCTGAGGGTTGGCTA	929	63
	5704F	F	TTTACGATGACGGACCGGAA	688	

Target virus	Primer name	Sense	Sequence 5’ → 3’	Product length (bases)	Annealing temp. (°C)
	6391R	R	TACCGCTCCTGAGCATATCCA		60
	6201F	F	AATTGGTGTCGAGGAGGACT		
	6844R	R	ATGAGAACGGGGCGTTGTAG	644	63
	6662F	F	ACATAACACGAAACTCGCGTC		
	7169R	R	TTCGACTCCCGGATAACCTGT	508	57
	6971F	F	AGCTGGTGGAAAAGCCCAAA		63
	7758R	R	TGGCTGCTACACCTCCTACA	788	
	7620F	F	GGACCAATTTCCGAAGTGGC		
	8229R	R	TTTCCAACCTGCCTGTGTGA	610	60
	8015F	F	TAACGGAGATGATTGGGGCG		
	8856R	R	TCGGTGTCTTGGTGTTTGCT	842	63
	8709F	F	TTCTTTGACTCAGGCGACCC		
	9263R	R	TTGCGGAGTGCCTATCATCC	555	63
	9040F	F	TTAACACCGACGGACCATCTC		
	9493R	R	TTCCGAAATTTTGCCGTAGTC	454	57
TR30069|c1	160F	F	TCCTGTGTCGGAGCAGTAGT	605	60.3
	764F	R	GGGTCCGGGATTGAGTTCAA		59.7
	587F	F	AGACTACCAACATCAGAACTATTGT	700	57.8
	1286R	R	GCTTGGTTGTCTATGTCGCA		58.6
	977F	F	GGGAGCAAAAACAAACAACGC	609	59.7
	1585R	R	TCAAACCAGCGGTCGATACC		60.1
	1422F	F	CGATGTGGGGAGTTAGGACG	646	59.9
	2067R	R	TCGGGGTTGATTGAGAATCCT		58.8
	1884F	F	ATGAGTTGGCCACTTGCAGT	667	60.2
	2250R	R	CGTCCGGTTCGAATCTTGTC		59
	2398F	F	TGGATCCTGAATCTGGAGCT	604	57.8
	3001R	R	TTCTCTCCCCACGTTTGCAA		59.8
	2798F	F	CGACGTTTTAGGACATGTAGTGG	686	59.4
	3483R	R	TCGGCTTTAAGGTGTTGGGG		60.3
	3313F	F	CAGCTGCATCGAACAAGCAA	635	59.8
TR30069|c0	16F	F	AGTATCCTGAGCCATGGGGG	631	60.8
	646R	R	GAAGGTGTGGGAAGCTCCAT		59.7
	445F	F	CAGGTGATCTCCTGTAGCCT	644	58.2
	1088R	R	ATGACGGACCGGAAAATGCT		60
	893F	F	GCCGCCTTAGTAGCACAGAT	459	59.9
	1351R	R	ACGAGTGTTCTCAAATGGACCA		59.9
TR31304|c0	20F	F	GACCAGGTTTGTTTGCCGAC	468	60
	487R	R	GACAGAGACCCGAACACCTG		60
	308F	F	TGCGGGATGTGAAAGAGCAT	663	60
	970R	R	AACGCCTTCGAATCCTTGGA		59.7
	869F	F	CAGGGTTGACCGAATGTACCA	699	60
	1567R	R	TCATGACCTAAAAGCGTCACCT		59.7
	1357F	F	TCCCACCATTGTTCCTTGAATCT	666	59.9
	2022R	R	CCCAAAACTCTTGTTCGGCAG		60
	1905F	F	TGTGCTACCCTTCTCAAAGCT	681	59.3
	2585R	R	CTTCTCCTCGCGTGTCAAGA		59.8
	2150F	F	CATGGCGTCGATTGAGGAGA	696	59.9
	2845R	R	GCCGAGTCCTTAACGGATGT		58.8
	2567F	F	CTTGACACGCGAGGAGAAGA	663	59.8
	3229R	R	GGGGCAACTGCAATCTTTCC		59.8
	2970F	F	AAGCGGAAGGTGGAAGCTTA	681	59.3
	3650R	R	CCACGCTTCTCTCAATCGGA		59.8
	3211F	F	GAAAGATTGCAGTTGCCCCC	663	59.8
	3873R	R	GTCGCAGCACCAACTCAAAG		60
	3669F	F	ATATCCGGTGATTGTGGGGC	357	59.9
	4025R	R	AGGGGCACTAGAGGGTTCAG	468	60.6

Supplementary Table S3: Argentine ant sampling locations for initial metatranscriptomic discovery / detection (and Sanger sequencing) and confirmatory RT-PCR of selected viruses.

Purpose	Location	Address	Coordinates	# ants
Metatranscriptomic discovery / detection and Sanger sequencing				
	**Northern**			
	Piha	North Piha Rd	-36.958250 174.469110	50
	Titirangi	Daffodil Street	-36.932020 174.652506	50
	Birkdale	Bishopgate Street	-36.801188 174.704871	50
	Total extracted			*150*
	**Southern**			
	Paraparaumu	Kenakena School	-40.882700 174.993715	20
	Picton	33 Scotland St	-41.296241 173.999328	20
	New Plymouth	36 Richmond Street	-35.046900 173.102520	20
	Christchurch	Riccarton High School	-43.535554 172.565062	20
	Petone	Jackson St	-41.223924 174.872861	20
	Gisborne	State Highway 35	-38.635600 178.156010	20
	Total extracted			*120*
Confirmatory RT-PCR				
	**Northern**			
	Dargaville	32 Gladston Road	-35.937810 173.871096	30
	Titirangi	Daffodil Street	-36.932020 174.652506	30
	Birkdale	Bishopgate Street	-36.801188 174.704871	24
	Total extracted			*84*
	**Southern**			
	Blenheim	35 Park Terrace	-41.512148 173.967678	30
	Paraparaumu	Kenakena School	-40.882700 174.993715	30
	New Plymouth	36 Richmond Street	-35.046900 173.102520	30
	Christchurch	Riccarton High School	-43.535554 172.565062	30
	Total extracted			*120*
	**Ants associated with bees**			
	Te Kao	Salt Road	-34.681220 173.001740	30
	Waipapa	Waipapa / West Coast Rd	-35.021770 173.216260	30
	Rangiputa	Puheke Hill	-34.867822 173.318306	30
	Total extracted			*90*

Supplementary Table S4: Primers for RT-PCR used to assay for viruses infecting bees and ants that were assessed in our study.

**Virus**	**Primer name**	**Sequence 5' → 3'**	**Product size (nt)**	**References**
Kashmir bee virus	KBV83F	ACCAGGAAGTATTCCCATGGTAAG	79	[1]
	KBV161R	TGGAGCTATGGTTCCGTTCAG		
	Tag-KBV83F	agcctgcgcaccgtggACCAGGAAGTATTCCCATGGTAAG		
Acute bee paralysis virus	ABV95F	TCCTATATCGACGACGAAAGACAA	65	[1]
	ABV159R	GCGCTTTAATTCCATCCAATTGA		
Chronic bee paralysis virus	CBPV304F	TCTGGCTCTGTCTTCGCAAA	68	[1]
	CBPV371R	GATACCGTCGTCACCCTCATG		
Black queen cell virus	BQCV8195F	GGTGCGGGAGATGATATGGA	71	[1]
	BQCV8265R	GCCGTCTGAGATGCATGAATAC		
	Tag-BQCV-sense	agcctgcgcaccgtggTCAGGTCGGAATAATCTCGA	420	[2]
	BQCV-antisense	GCAACAAGAAGAAACGTAAACCAC		
Deformed wing virus	DWV-F8668	TTCATTAAAGCCACCTGGAACATC	136	[3]
	DWV-B8757	TTCATTAAAGCCACCTGGAACATC		
	Tag-F15	agcctgcgcaccgtggTCCATCAGGTTCTCCAATAACGGA		
	B23	CCACCCAAATGCTAACTCTAAGCG		
Linepithema humile virus 1	n6409-A656F	AGGAAACGCAGCCAGTGCTATT	399	[4]
	n6409-A1055R	GTCACCTGACTCCTTGCCTGATTT		
Israeli acute paralysis virus	IAPV_F	CGAACTTGGTGACTTGAAGG	114	[5, 6]
	IAPV_R	ACGTCAGTCGTCTTCCAGGT		
Solenopsis invicta virus 1	p517	CAATAGGCACCAACGTATATAGTAGAGATTGGA	254	[7]
	P519	GGAATGGGTCATCATATAGAAGAATTG		
Formica exsecta virus 1	Fex1_7681F	ACCGCTGCCAATGGTATTGA	106	Our study
	Fex1_7786R	TTGCTGCTACGCTACCTACG		
Varroa destructor virus 1	VDVqPCR-for	TGGCTAATCGACGTAAAGCA	200	[8]
	VDVqPCR-rev	ACTAATCTCTGAGCCAACACGT		
	Tag-VDVqF	agcctgcgcaccgtggTGGCTAATCGACGTAAAGCA		
All primers	Tag	agcctgcgcaccgtgg		[9]

Supplementary Table S5: Alignment used to build the phylogenetic trees presented in Figures 4 and 5 (Microsoft Excel spreadsheet).

References

1. Chantawannakul, P., Ward, L., Boonham, N. & Brown, M. A scientific note on the detection of honeybee viruses using real-time PCR (TaqMan) in *Varroa* mites collected from a Thai honeybee (*Apis mellifera*) apiary. *J. Invertebr. Pathol.* **91**, 69-73, doi:http://dx.doi.org/10.1016/j.jip.2005.11.001 (2006).
2. Peng, W., Li, J., Boncristiani, H., Strange, J. P., & Hamilton, M. Host range expansion of honey bee *black queen cell virus* in the bumble bee, *Bombus huntii*. *Apidologie*. **42**, 650, http://doi.org/10.1007/s13592-011-0061-5 (2011).
3. Yañez, O., Jaffé, R., Jarosch, A., Fries, I., & Moritz, R. *Deformed wing virus* and drone mating flights in the honey bee (*Apis mellifera*): implications for sexual transmission of a major honey bee virus. *Apidologie*. **43**, 17, http://doi.org/10.1007/s13592-011-0088-7 (2012).
4. Sébastien, A. *et al.* Invasive ants carry novel viruses in their new range and form reservoirs for a honeybee pathogen*. Biol. Lett*. **11** (2015).
5. Martin, S. J. *et al*. Global honey bee viral landscape altered by a parasitic mite. *Science* **336**, 1304-1306, doi:10.1126/science.1220941 (2012).
6. Palacios, G. *et al.* Genetic Analysis of *Israel Acute Paralysis Virus*: Distinct clusters are circulating in the United States. *J. Virol*. **82**, 6209-6217, doi:10.1128/jvi.00251-08 (2008).
7. Hashimoto, Y., Valles, S. M. & Strong, C. A. Detection and quantitation of *Solenopsis invicta virus* in fire ants by real-time PCR. *J. Virol. Methods*. **140**, 132–139 (2007)
8. Zioni, N., Soroker, V., & Chejanovsky, N. Replication of *Varroa destructor virus 1* (VDV-1) and a *Varroa destructor virus 1-deformed wing virus* recombinant (VDV-1-DWV) in the head of the honey bee. *Virology*, **417**(1), 106–112. http://doi.org/10.1016/j.virol.2011.05.009 (2011).
9. Yue, C., & Genersch, E. RT-PCR analysis of *Deformed wing virus* in honeybees (*Apis mellifera*) and mites (*Varroa destructor*). *J. Gen. Virol*., **86**, 3419–3424. http://doi.org/10.1099/vir.0.81401-0 (2005).
